# Formation of *Potato Virus A*-Induced RNA Granules and Viral Translation Are Interrelated Processes Required for Optimal Virus Accumulation

**DOI:** 10.1371/journal.ppat.1005314

**Published:** 2015-12-07

**Authors:** Anders Hafrén, Andres Lõhmus, Kristiina Mäkinen

**Affiliations:** Department of Food and Environmental Sciences, Viikki Plant Science Centre, University of Helsinki, Helsinki, Finland; Agriculture and Agri-Food Canada, CANADA

## Abstract

RNA granules are cellular structures, which play an important role in mRNA translation, storage, and degradation. Animal (+)RNA viruses often co-opt RNA granule proteins for viral reproduction. However, the role of RNA granules in plant viral infections is poorly understood. Here we use *Potato virus A* (PVA) as a model potyvirus and demonstrate that the helper component-proteinase (HCpro), the potyviral suppressor of RNA silencing, induces the formation of RNA granules. We used confocal microscopy to demonstrate the presence of host RNA binding proteins including acidic ribosomal protein P0, argonaute 1 (AGO1), oligouridylate-binding protein 1 (UBP1), varicose (VCS) and eukaryotic initiation factor iso4E (eIF(iso)4E) in these potyvirus-induced RNA granules. We show that the number of potyviral RNA granules is down-regulated by the genome-linked viral protein (VPg). We demonstrated previously that VPg is a virus-specific translational regulator that co-operates with potyviral RNA granule components P0 and eIF(iso)4E in PVA translation. In this study we show that HCpro and varicose, components of potyviral RNA granules, stimulate VPg-promoted translation of the PVA, whereas UBP1 inhibits this process. Hence, we propose that PVA translation operates via a pathway that is interrelated with potyviral RNA granules in PVA infection. The importance of these granules is evident from the strong reduction in viral RNA and coat protein amounts that follows knock down of potyviral RNA granule components. HCpro suppresses antiviral RNA silencing during infection, and our results allow us to propose that this is also the functional context of the potyviral RNA granules we describe in this study.

## Author Summary

Cytoplasmic RNA granules play a central role in mRNA metabolism both in animal and plant cells. Here we demonstrate that *Potato Virus A* (PVA)-encoded multifunctional protein HCpro induces RNA granules, which contain in addition to HCpro several plant RNA granule proteins. We have earlier described a potyvirus-specific translational pathway where PVA-encoded VPg boosts PVA RNA accumulation and translation. In the current study, we show that many of the RNA granule proteins participate in potyviral translation and conclude that VPg-mediated active viral translation and formation of HCpro-induced RNA granules are interrelated processes. This mechanism is required to overcome active RNA silencing and to achieve optimal viral gene expression and virus accumulation.

## Introduction

A feature associated with mRNA in eukaryotic cells is its ability to assemble into ribonucleoprotein (RNP) complexes, which in turn may form large RNA granules. The best characterized RNA granules in yeast and animal cells are processing bodies (PBs) and stress granules (SGs) [1–3]. Over 40 different proteins have been identified both in SGs and PBs and many of them are shared between different RNA granule types in yeast and mammals [4]. The composition of RNA granules is currently far less studied in plants. Nevertheless, plant SGs are characterized by several hallmark-proteins like oligouridylate-binding protein 1 (UBP1), eukaryotic initiation factor 4E (eIF4E) and poly-A binding protein (PABP) whereas plant PBs contain decapping proteins 1 and 2 (DCP1 and DCP2), WD-domain protein varicose (VCS) and argonaute 1 (AGO1) [2,3,5]. A model termed “the mRNA-cycle”, in which mRNAs are sorted between translation, storage, and degradation in a process involving ribosomes, SGs, and PBs, has been set forth [6]. Processes that regulate mRNA expression within these granules are mRNA decay, RNA silencing and translational repression. Furthermore, the mechanisms of mRNA decay and RNA silencing are coupled functionally [7,8], and also show spatial overlap with the RNA silencing proteins AGO1 and silencing defective 3 (SDE3) localized in *Arabidopsis* PBs [9].

Pathways involved in host mRNA regulation also have a role in the coordination of viral RNA (vRNA) expression. Many (+) RNA animal viruses manipulate PBs and SGs by directing essential RNA granule components to novel locations and co-opting them to virus-specific processes [10]. *Brome mosaic virus* (BMV; genus Bromovirus) requires PB components for replication and translation of its RNA in yeast, stressing the importance of RNA granules in plant virus infection [11]. Furthermore, BMV RNA is located to PBs in a process dependent on specific vRNA elements [12]. Antiviral RNA silencing plays a critical role in restricting plant viral infections and is usually counteracted by viral suppressors of RNA silencing (VSRs) [13]. Despite many interesting discoveries on how VSRs act to suppress RNA silencing it is still unknown how pathways of vRNA translation, antiviral RNA silencing and its suppression converge in plants, and how different RNA granules and their components are involved in these processes.

We previously demonstrated that viral protein genome-linked (VPg), together with acidic ribosomal protein P0 and eIF4E/eIF(iso)4E, promotes the translation of *Potato virus A* (PVA; genus *Potyvirus*) RNA [14,15]. In the current study we show that PVA induces RNA granules which contain the VSR HCpro, PVA RNA, P0, eIF(iso)4E and plant proteins linked to RNA granules and silencing. We show that most of the identified granule components are required to achieve a robust infection, as demonstrated earlier for P0 and eIF4E / eIF(iso)4E [15], underscoring their pro-viral role. We demonstrate that formation of these granules is induced by HCpro and, furthermore, that high concentrations of VPg results in their disappearance. The granule components P0, VCS and eIF(iso)4E are stimulators, whereas UBP1 is a negative regulator of VPg- activated translation, suggesting that PVA-induced RNA granules are dynamically coupled to VPg-activated PVA RNA translation.

## Results

### PVA viral suppressor of RNA silencing, HCpro, induces RNA granules

**Fig 1:**
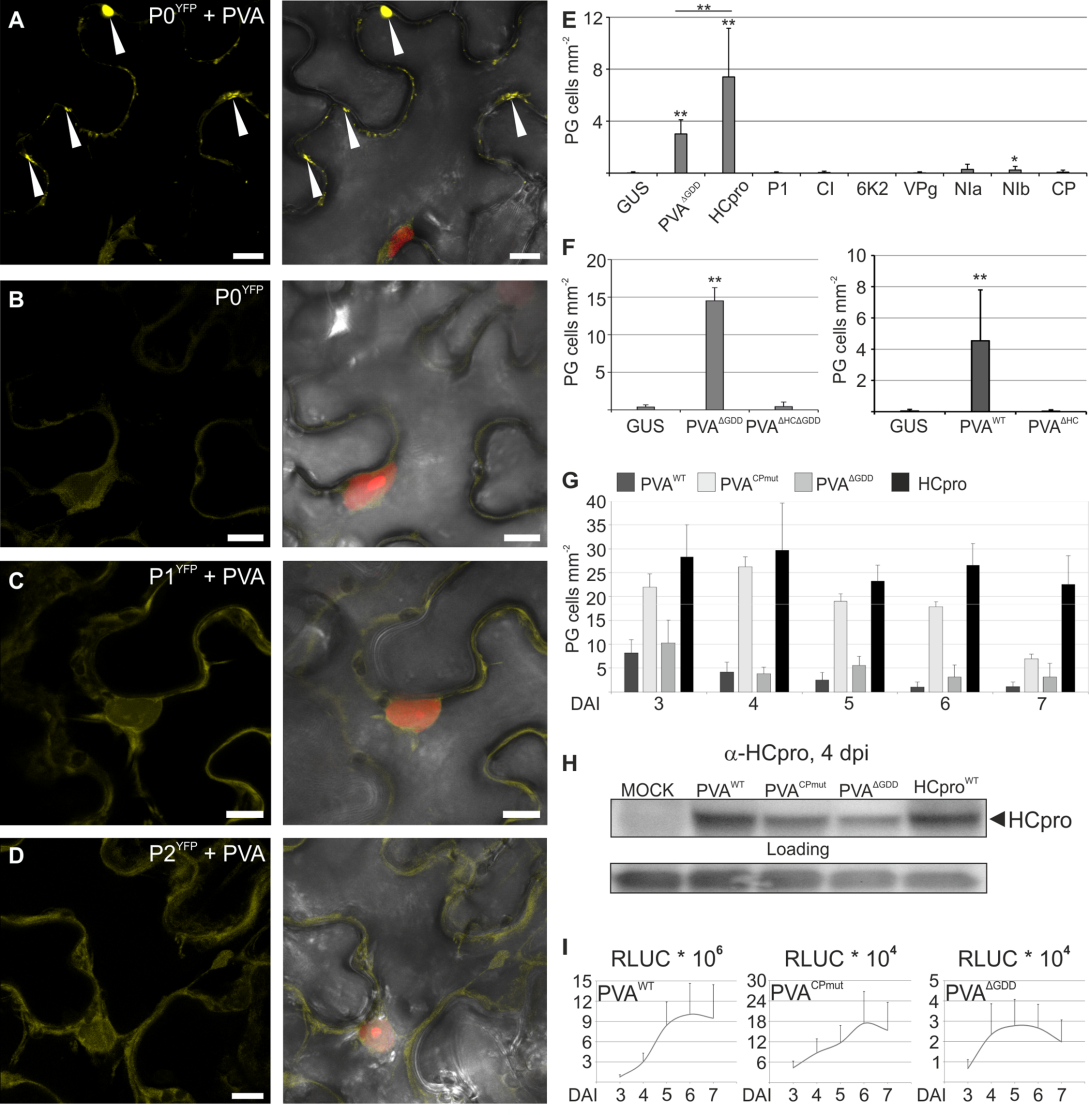
HCpro, the potyviral suppressor of RNA silencing, is the protein responsible for PVA-induced granules (PGs). P0^YFP^ was co-expressed with PVA^WT^ (A) and alone (B), and P1^YFP^ (C) and P2^YFP^ (D) were expressed with PVA^WT^ in *Nicotiana benthamiana* leaves using agroinfiltration and imaged by confocal microscopy three days later. RFP was expressed in A-D to visualize the nucleus. In (A- D) the left panel shows the YFP signal and the right panel an overlay view of YFP, nuclear RFP and the bright field (BF) as Z-stack projections. Arrowheads point out P0^YFP^-labeled PGs in (A). Scale bar; 10 μm. (E) Frequency of cells/mm^2^ containing PGs in leaves expressing P0^YFP^ together with PVA proteins P1, HCpro, CI, 6K2, VPg, NIa, NIb or CP. PVA^ΔGDD^ was expressed as a positive and GUS as a negative control for PG induction. (F) Frequency of cells/mm^2^ containing PGs in leaves expressing P0^YFP^ together with GUS, PVA^ΔGDD^ or PVA^ΔHCΔGDD^ (left panel) or GUS, PVA or PVA^ΔHC^ (right panel). (G) Frequency of cells/mm^2^ showing PGs in leaves expressing P0^YFP^ with PVA^WT^, PVA^CPmut^, PVA^ΔGDD^ or HCpro plotted as a function of time, (H) a western blot analysis of HCpro accumulation in PVA^WT^, PVA^CPmut^, PVA^ΔGDD^ and HCpro samples using anti-HCpro PAb four days post infiltration and (I) virus derived RLUC activities analyzed in the same samples as in (H), again plotted as a function of time (days post infiltration). Modified PVA infectious cDNA constructs used in this study are schematically presented in (S1 Fig). All quantitative data is presented as means and the error bars indicate the standard deviations. (p < 0.01 **, p < 0.05 *).

Based on our previous finding that the acidic ribosomal protein P0 promotes PVA translation [15], we analyzed the cellular distribution of P0 in infected cells. We fused P0 to YFP (P0^YFP^) and imaged the protein in the presence and absence of PVA. In the presence of wild type PVA (PVA^WT^), P0^YFP^ formed cytoplasmic granule-like structures (Fig 1A). Such granules were rarely observed in healthy cells (Fig 1B), and thus we refer to them as PVA-induced granules (PGs). Neither of the two other ribosomal stalk proteins fused to YFP, P1^YFP^ or P2^YFP^, localized to PGs (Fig 1C and 1D), suggesting that PGs were mainly labeled by non-ribosomal P0. P0 promotes PVA infection [15], which we also observed with P0^YFP^ (S2A Fig) verifying that the P0^YFP^ fusion protein could participate in PVA infection. We also observed that P0^YFP^ accumulated to higher levels in the presence of PVA RNA than in its absence (S2B and S2C Fig).

We next studied whether viral replication or particle assembly and movement, affected PG formation by using two PVA mutants. PVA^CPmut^ carries a mutation in the capsid protein (CP) that disrupts particle assembly and viral cell-to-cell and systemic movement, whereas PVA^∆GDD^ is impaired in replication due to a mutation in its RNA-dependent RNA polymerase (RdRp) [16]. Both mutants induced PGs (S2D Fig), showing that these processes were not required for PG formation.

To investigate whether PG induction was caused by a viral protein, we co-expressed PVA proteins with P0^YFP^ and calculated the frequency of cells showing PGs. PGs were induced by HCpro, while other tested PVA proteins did not induce PGs above the level of the negative control GUS (Fig 1E). When the HCpro gene was deleted from the PVA^ΔGDD^ (PVA^ΔHCΔGDD^) or PVA^WT^ (PVA^ΔHC^), no PGs were detected confirming that HCpro was essential for PG induction (Fig 1F).

A time course analysis showed that the number of cells containing PGs induced by HCpro and PVA^CPmut^ was substantially higher than that induced by PVA^WT^ or PVA^∆GDD^ (Fig 1G), despite higher levels of HCpro during PVA^WT^ than PVA^CPmut^ infection (Fig 1H). PVA and its mutants carried *Renilla* luciferase (*RLUC*) gene within the polyprotein encoding ORF. RLUC activity could therefore quantitatively report the level of viral gene expression [16]. As expected, the highest viral gene expression was derived from PVA^WT^ (Fig 1I), as also seen from HCpro protein amount (Fig 1H). The lack of correlation between the amounts of HCpro and PGs during PVA^WT^ infection suggested that more factors than HCpro concentration alone regulate the intracellular trajectories of PGs. Virion assembly could be an example of a process that reduces PG formation. A general observation was that the amount of cells showing PGs was somewhat variable between experiments, but the differences between treatments and constructs within experiments were always reproducible.

### HCpro and common RNA granule components localize to PGs

**Fig 2:**
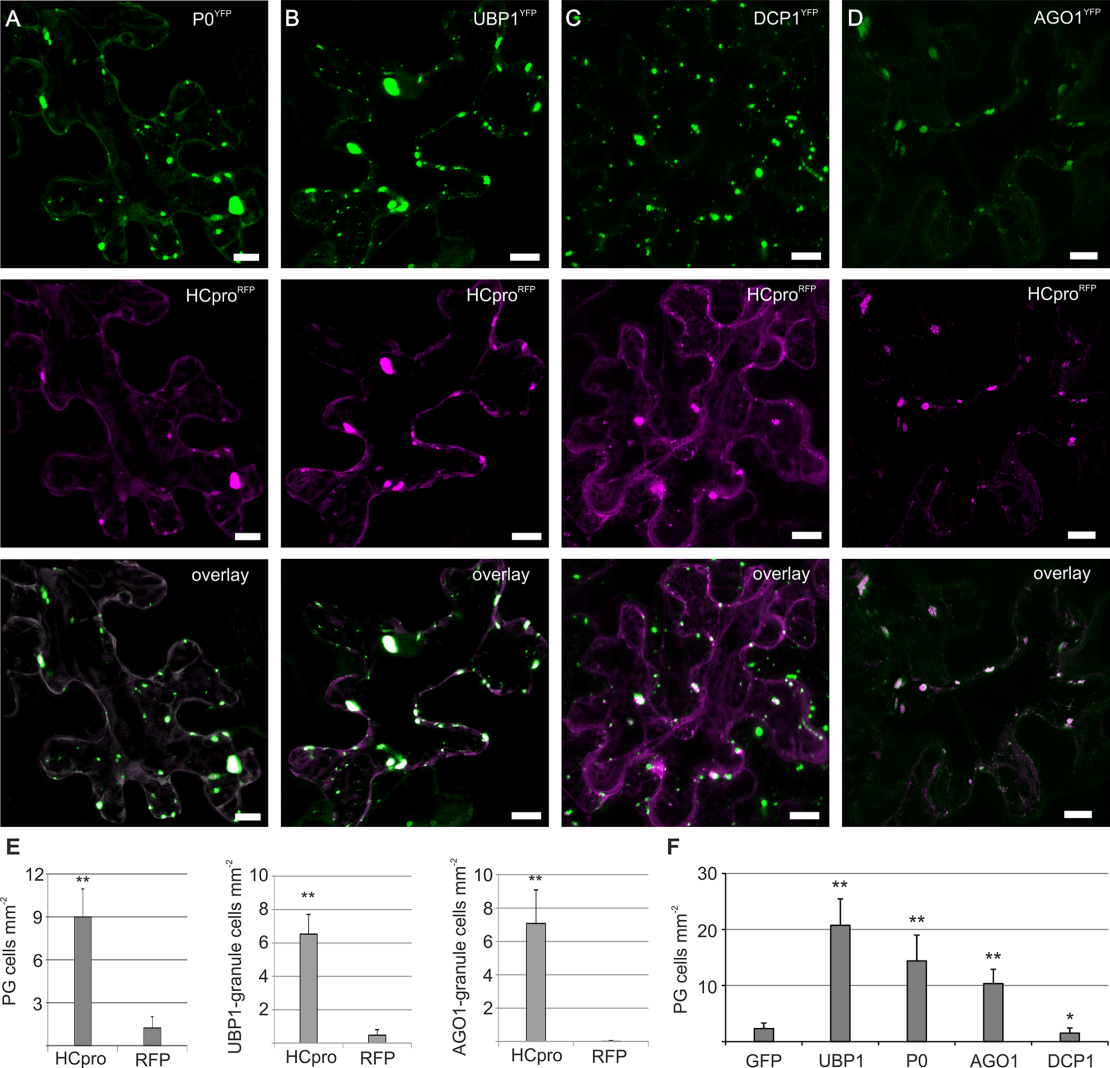
HCpro and several host proteins involved in cellular RNA metabolic processes associate with PGs. HCpro^RFP^ was used to induce PGs (A-D). Host proteins fused to fluorescing proteins were co-expressed with HCpro^RFP^ as indicated using agroinfiltration of *N*. *benthamiana* leaves (using OD_600_ 0.1 for each construct), and their localization was examined by confocal microscopy three days later. HCpro^RFP^ co-expressed with P0^YFP^ (A), UBP1^YFP^ (B), DCP1^CFP^ (C) and AGO1^CFP^ (D). The images are Z-stack projections to clearly show the absence or presence of PGs in the cell and signal overlaps were verified from single layer images. Scale bar; 10 μm. (E) The frequency of cells/mm^2^ showing granule structures labeled by P0^YFP^, UBP1^YFP^ and AGO1^CFP^ was compared when these host proteins were co-expressed with either HCpro^RFP^ or RFP alone (see also S3 Fig). Since DCP1 granules (PBs) are constantly present in all cells regardless of HCpro, these data are not given for DCP1-granules. (F) The frequency of cells/mm^2^ showing HCpro granules when granule components UBP1, P0, AGO1 and DCP1 were co-expressed using high *Agrobacterium* concentrations (OD_600_ 0.5). GFP was expressed as control. (p < 0.01 **, p < 0.05 *)

Next we asked whether HCpro is a structural component of PGs. PGs were induced by co-expressing P0^YFP^ with HCpro^RFP^ and imaging revealed an extensive overlap of HCpro^RFP^ and P0^YFP^ signals in PGs (Fig 2A). This was neither observed for P0^YFP^ and non-fused RFP (S3A Fig), nor HCpro^RFP^ and non-fused YFP (S3B Fig), demonstrating that HCpro is a component of PGs.

We hypothesized that PGs could be RNA granules and set out to image HCpro together with known plant RNA granule markers. TIA-1 is a common but not exclusive marker of animal and yeast SGs, and it is a homolog of plant UBP1. UBP1 labels plant SGs during heat and oxidative stress [2,5]. In the absence of HCpro, UBP1^YFP^ localized mainly to nucleus (S3C Fig), but when expressed together with HCpro^RFP^, UBP1^YFP^ localized abundantly to PGs (Fig 2B), showing that HCpro relocated UBP1 to granules similarly as observed for P0. HCpro^RFP^ granules commonly appeared bigger in size when co-expressed with either P0^YFP^ or UBP1^YFP^ compared to free YFP, suggesting that P0 and UBP1 could promote HCpro aggregation. We applied heat stress to induce SGs, and monitored P0^YFP^ and UBP1^YFP^ granulation (S3G and S3H Fig). The amount of cells showing UBP1 labeled granules increased upon heat treatment. However, heat stress did not induce P0^YFP^-containing PGs, suggesting that P0 is not a component of heat-induced RNA granules.

DCP1 and AGO1 are components of plant PBs [3,17]. We found that DCP1 co-localized to some extent and AGO1 fully with HCpro^RFP^ in PGs (Fig 2C and 2D). In accordance with the constitutive nature of PBs, DCP1^CFP^ labeled granules were present in most cells irrespective of HCpro expression (S3D Fig). Importantly, AGO1 did not localize to granule-like structures in the absence of HCpro (S3 Fig). Similarly, eIF(iso)4E only localized to PGs in the presence of PVA (S3F Fig).

The frequency of cells/mm^2^ showing P0-, UBP1- and AGO1-granules was compared when these host proteins were co-expressed either with HCpro^RFP^ or RFP (Fig 2E), showing that granules were formed predominantly in the presence of HCpro. Moreover we found that the frequency of cells showing HCpro^RFP^ granules increased significantly during strong over-expression of UBP1, P0 and AGO1 but not DCP1 (Fig 2F). Here we used higher *Agrobacterium* concentrations (OD_600_ 0.5) than in the localization experiment, and the result suggests that UBP1, P0 and AGO1 promote HCpro granule formation. We conclude that several known markers of plant RNA granules can be localized to HCpro-induced PGs, and the compositional divergence suggests that the structures could be non-canonical derivatives of SGs and or PBs.

### PGs are RNA granules and contain viral RNA

**Fig 3:**
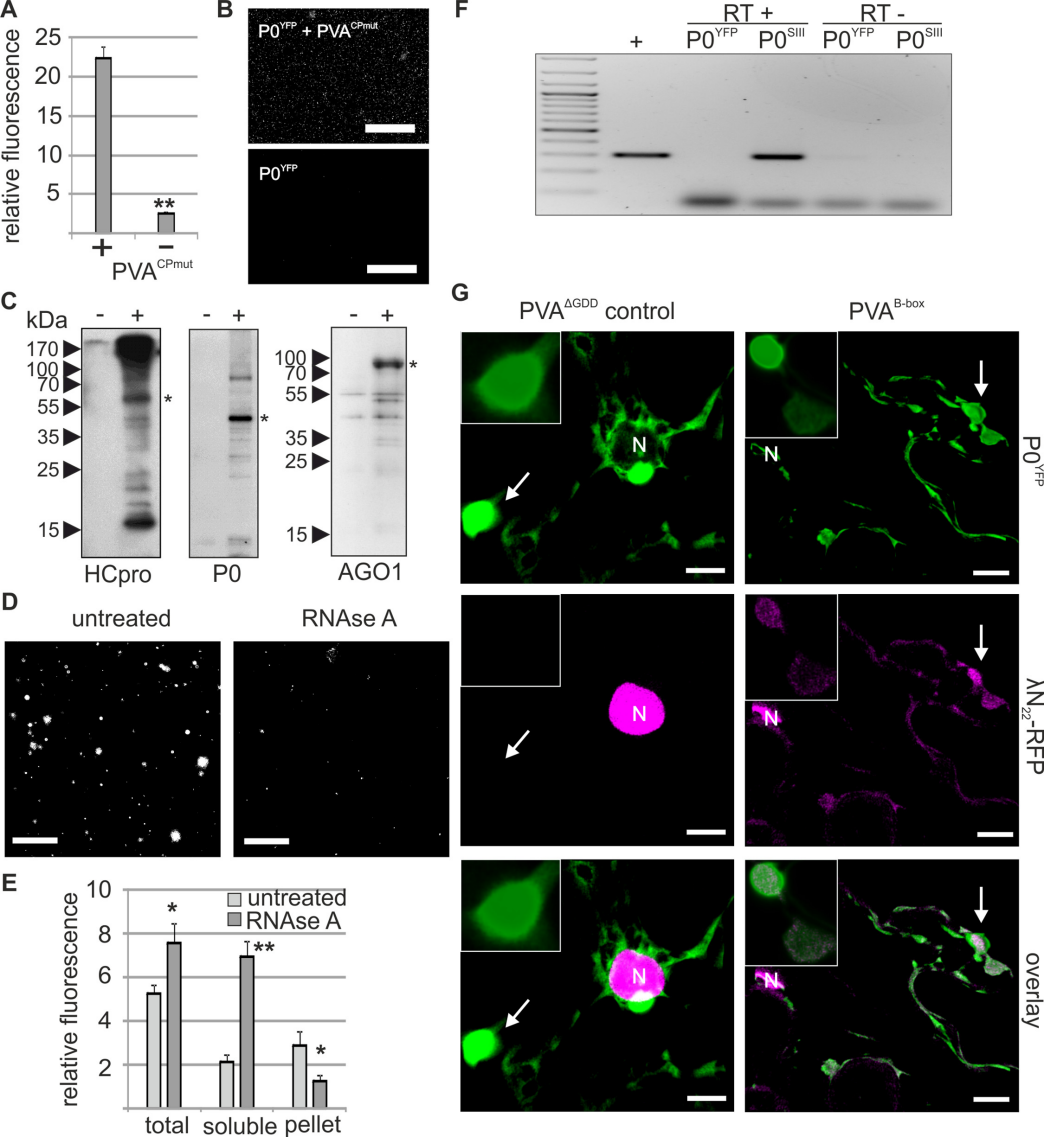
PGs are RNA granules to which also PVA RNA can localize. (A) Fractions containing PGs labeled with P0^YFP^ were isolated from PVA^CPmut^ infected leaf lysates (+ sample), as described in materials and methods. Lysates from non-infected leaves expressing P0^YFP^ were fractionated similarly (- sample). Three parallel fractions of each sample type were used for fluorescence quantification and relative fluorescence units are given as a mean ± standard deviation. (B) Fractions quantified in (A) were imaged using epifluorescence microscopy to show successful capture of PVA-induced PGs. Scale bar; 500 μm. (C) PG fractions were isolated from mock-infiltrated leaves (-) and PVA^CPmut^ RNA-expressing leaves (+) at 3 DAI and subjected to a western blot detection of endogenous HCpro, P0 and AGO1. The asterisks denote the expected position of the corresponding protein. (D) Similar samples enriched for PGs as in (A and B), were incubated with or without RNAse A and imaged using epifluorescence microscopy. Scale bar; 20 μm. (E) RNAse A-mediated release of fluorescence from isolated PGs (D) was quantified by analyzing fluorescence in total, soluble and low-speed pellet fractions after RNAse A treatment compared to control samples (n = 3). (F) PG fractions were prepared from leaves expressing PVA together with either P0^YFP^ (control) or Strep-III-tagged P0 (P0^SIII^), and subjected to Strep-tag based affinity purification. RNA was isolated from the affinity-purified samples and subjected to reverse transcription (RT+), followed by PCR detection of viral RNA. Total RNA from PVA infected leaves was used as a positive control for RT-PCR. The RT-minus control (RT-) was negative. (G) Bacteriophage λ B-box RNA elements were fused to the 3´ UTR within PVA^∆GDD^ icDNA (PVA^B-box^; S1 Fig). Binding of λN22^RFP^ to the B-box RNA element enabled visualization of PVA^B-box^ RNA *in vivo*. PGs were induced either by PVA^∆GDD^ (control) or PVA^B-box^ and visualized by P0^YFP^ (green channel), and λN22^RFP^ was co-expressed to label B-box RNA. The RFP signal was mainly found in nuclei due to the nuclear localization signal present in λN22^RFP^, but also in the cytoplasm and PGs in the presence of PVA^B-box^ (magenta channel). The images are projections of Z-stacks with a single layer inset from the area indicated with an arrow. Scale bar; 10 μm.

To study the composition of PGs further, we developed a sucrose density-based centrifugation protocol to enrich PGs and prepared samples from leaves expressing P0^YFP^ alone or together with the inducer of PGs, PVA^CPmut^. The P0^YFP^ fluorescence concentrated on the 80% sucrose layer under the conditions favoring PG formation and this fraction was collected for fluorescence quantification. The relative amount of P0^YFP^ fluorescence was almost 10 times higher in the sample containing PVA than in the corresponding non-infected sample (Fig 3A). When these samples were compared using fluorescence microscopy, enrichment of PVA-induced PGs was evident (Fig 3B). This was further supported by western blot analysis showing accumulation of HCpro together with endogenous P0 and AGO1 in a PVA^CPmut^-induced PG fraction while no accumulation was detected in the corresponding control fraction (Fig 3C). A substantial amount of HCpro was involved in a high molecular weight complex in addition to the band migrating in the correct size range, and the additional faster migrating bands detected by AGO1 antibody could represent non-specific or degradation products. The antibody used to detect P0 also recognize P1, P2 and P3 proteins of the ribosomal stalk around 15 kDa in size, and the strong enrichment of specifically P0 suggests that its presence in these fractions is not coming from ribosomes, as already implied in (Fig 1).

While developing isolation conditions, we found that PGs tolerated both non-ionic detergents and high salt concentration, but incubation with RNAse A disrupted the isolated PGs (Fig 3D). When the amount of fluorescence in the soluble and low-speed pellet fractions of the PG samples was quantified before and after RNAse A treatment, we found that the amount of fluorescence in the low-speed pellet containing the visible PGs decreased after RNAse A treatment, with a concomitant increase of fluorescence in the soluble fraction (Fig 3E). This is analogous to what was observed for isolated PBs [18] and showed that RNA is a critical structural component of PGs.

PGs contain RNA and are induced by PVA, which implies that viral RNA could reside within PGs. To address this, we subjected PG fractions to affinity purification via Strep-III tag fused to P0 (P0^SIII^), followed by RNA extraction and reverse-transcription (RT)-PCR to detect viral RNA. Viral RNA was specifically detected in the P0^SIII^-sample, suggesting that it may reside in PGs (Fig 3F). We then analyzed the localization of viral RNA in relation to PGs utilizing the affinity of the λN22-peptide for an RNA element termed the B-box [19]. B-box RNA elements were fused to PVA^∆GDD^ (PVA^B-box^) to allow imaging of viral RNA with λN22-peptide fused to a fluorescent protein. λN22^RFP^ has a nuclear localization signal and is only retained in the cytoplasm upon binding to cytoplasmic B-box-labeled RNAs [19]. PVA^B-box^ was co-expressed with P0^YFP^ and λN22^RFP^ followed by imaging at 3 DAI (Fig 3G). We observed labeling of PGs only in a fraction of cells expressing both PGs and the RNA marker. Such low frequency of cells showing labeling has been reported when imaging SG-association with mRNAs using a similar approach, and labeling was speculated to depend on high enough expression of the imaged mRNA [20]. Nevertheless, when co-expressed with PVA^B-box^, λN22^RFP^ occasionally labeled both PGs and the cytoplasm, whereas λN22^RFP^ was exclusively detected in nuclei when co-expressed with control PVA^∆GDD^ devoid of B-box RNA elements. These data indicate that RNA is a structural component of PGs and that viral RNA can localize to PGs. PGs likely contain other RNAs as well since they are induced by HCpro alone, and complete RNA profiling of PGs is a future interest. Importantly, endogenous P0 and AGO1 accumulated in PG fractions, supporting that PG localizations of transiently expressed AGO1^CFP^ and P0^YFP^ as seen by confocal microscopy (Fig 2), are not artefacts of over-expression.

### PG formation is specific to HCpro among several viral RNA silencing suppressors

**Fig 4:**
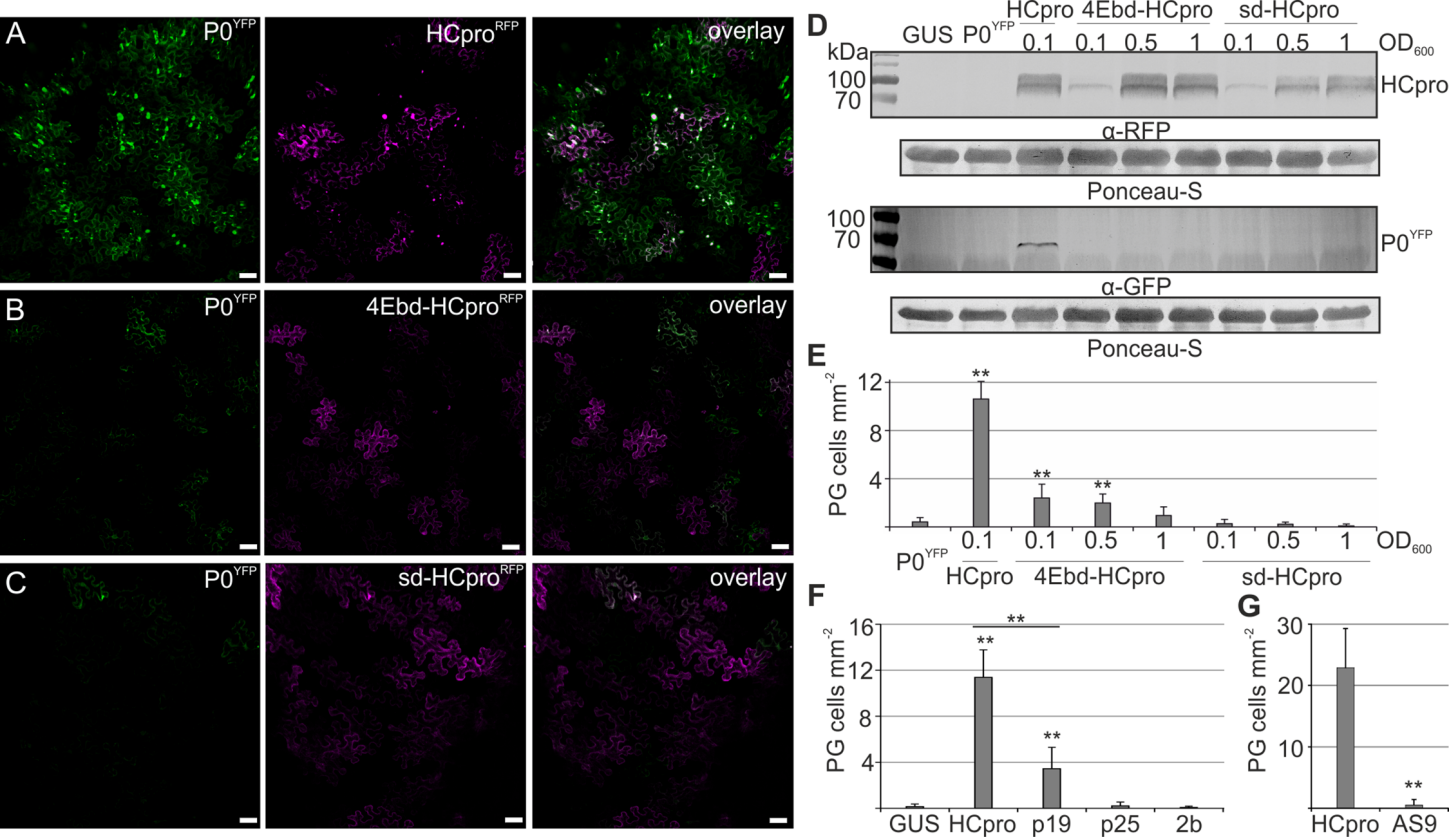
HCpro mutants lacking RNA silencing suppression and eIF4E binding are deficient in PG induction. P0^YFP^ was co-expressed with HCpro^RFP^ (A), 4Ebd-HCpro^RFP^ (B) or sd-HCpro^RFP^ (C) in *N*. *benthamiana* leaves using agroinfiltration. P0^YFP^ and HCpro^RFP^ signals were detected by confocal microscopy at 3 DAI and presented as Z-stack projections covering an area of multiple epidermal cells to clearly convey the frequency of PGs. Scale bar; 50 μm. (D) In order to achieve comparable levels of native HCpro^RFP^ and the mutants, 4Ebd-HCpro^RFP^ and sd-HCpro^RFP^ expression was increased by increasing the *Agrobacterium* concentration used in infiltrations (indicated by the OD_600_ values). P0^YFP^ was co-expressed with these. Protein accumulation was followed by western blot analysis using anti-RFP for HCpro and anti-GFP for P0 detection. (E) The frequency of cells/mm^2^ showing PGs was calculated using fluorescence microscopy in parallel with analyzing the protein levels in (D). (F) Frequency of cells/mm^2^ showing P0-labeled granules during co-expressed of P0^YFP^ with plant viral VSRs HCpro, P19, 2b and P25. (G) Frequency of cells/mm^2^ showing YFP-tagged TuMV HCpro or HCpro AS9 in PG-like structures. The PG frequencies are presented as means and the error bars indicate the standard deviations. (p < 0.01 **, p < 0.05 *).

HCpro is the potyviral suppressor of RNA silencing and a component of PGs, suggesting that PGs could have a link to RNA silencing. To test this hypothesis we introduced amino acid substitutions into HCpro that disrupt the RNA silencing suppression activity (silencing-deficient (sd)-HCpro) [21] and decrease the affinity to eIF(iso)4E (4E-binding deficient (4Ebd)-HCpro) [22]. Both mutants were fused to RFP, co-expressed with P0^YFP^ and imaged. Neither of the mutants induced PGs in a similar manner to the native HCpro (compare Fig 4A, 4B and 4C). Only a few small PGs containing 4Ebd-HCpro were observed, whereas sd-HCpro did not induce any detectable granulation.

Western blot analysis of the HCpro^RFP^ mutants indicated that both of them accumulated less than native HCpro^RFP^, perhaps because of their compromised RNA silencing suppression capacity, and no enhanced P0^YFP^ accumulation was detected in their presence (S4A Fig). The expression levels of 4Ebd- and sd-HCpro were raised to the level of the native HCpro by infiltrating higher concentrations of *Agrobacterium* carrying the mutant HCpro genes (Fig 4D). The frequency of cells showing PGs did not increase and was diminutive when compared to native HCpro induction (Fig 4E). This showed that the HCpro mutants failed to induce PGs irrespective of the mutant protein accumulation levels.

We quantified the capacity of HCpro^RFP^, 4Ebd-HCpro^RFP^ and sd-HCpro^RFP^ to suppress RNA silencing. A double-stranded RNA molecule was expressed to target *RLUC* mRNA, and the strength of silencing suppression was quantified by restored RLUC activity. As expected, sd-HCpro did not suppress RNA silencing and suppression by 4Ebd-HCpro was compromised (S4B Fig). Since both mutants concomitantly lost their capacity to suppress RNA silencing and to induce PGs, we are unable to conclude if the RNA silencing suppression is central for HCpro-mediated PG induction. Therefore we addressed whether PGs form in response to other viral suppressors of RNA silencing (VSRs) by expressing different VSRs with P0^YFP^ and analyzing the frequency of cells showing PG-like structures. Tombusviral P19 induced PG-like structures in a higher number of cells than the negative GUS control but with a clearly lower frequency than HCpro, whereas potexviral P25 and cucumoviral 2b did not induce PGs at all (Fig 4F). We verified the silencing suppression activity of the used VSRs. All VSRs could suppress hairpin RNA triggered silencing, P19 being the strongest suppressor (S4C Fig), whereas GUS, VPg and P0 could not. Finally, to verify that PG induction is a general potyviral HCpro function, we expressed YFP-tagged HCpro of *Turnip mosaic virus* (TuMV; genus *Potyvirus*) and calculated the frequency of cells showing the protein in granules (Fig 4G). TuMV HCpro localized abundantly in PG-like structures, suggesting that this phenomenon is common to potyviruses. Furthermore, a silencing deficient mutant of TuMV HCpro (AS9) [23] failed to induce any PGs (Fig 4G). This was expected because the mutation in AS9 is similar to that in sd-HCpro of PVA To summarize, PG formation is not a general response to suppression of RNA silencing but rather specific to potyvirus HCpro, and whether silencing suppression activity is required for HCpro to induce granules remains currently open.

### PG components and VPg co-regulate vRNA translation

**Fig 5:**
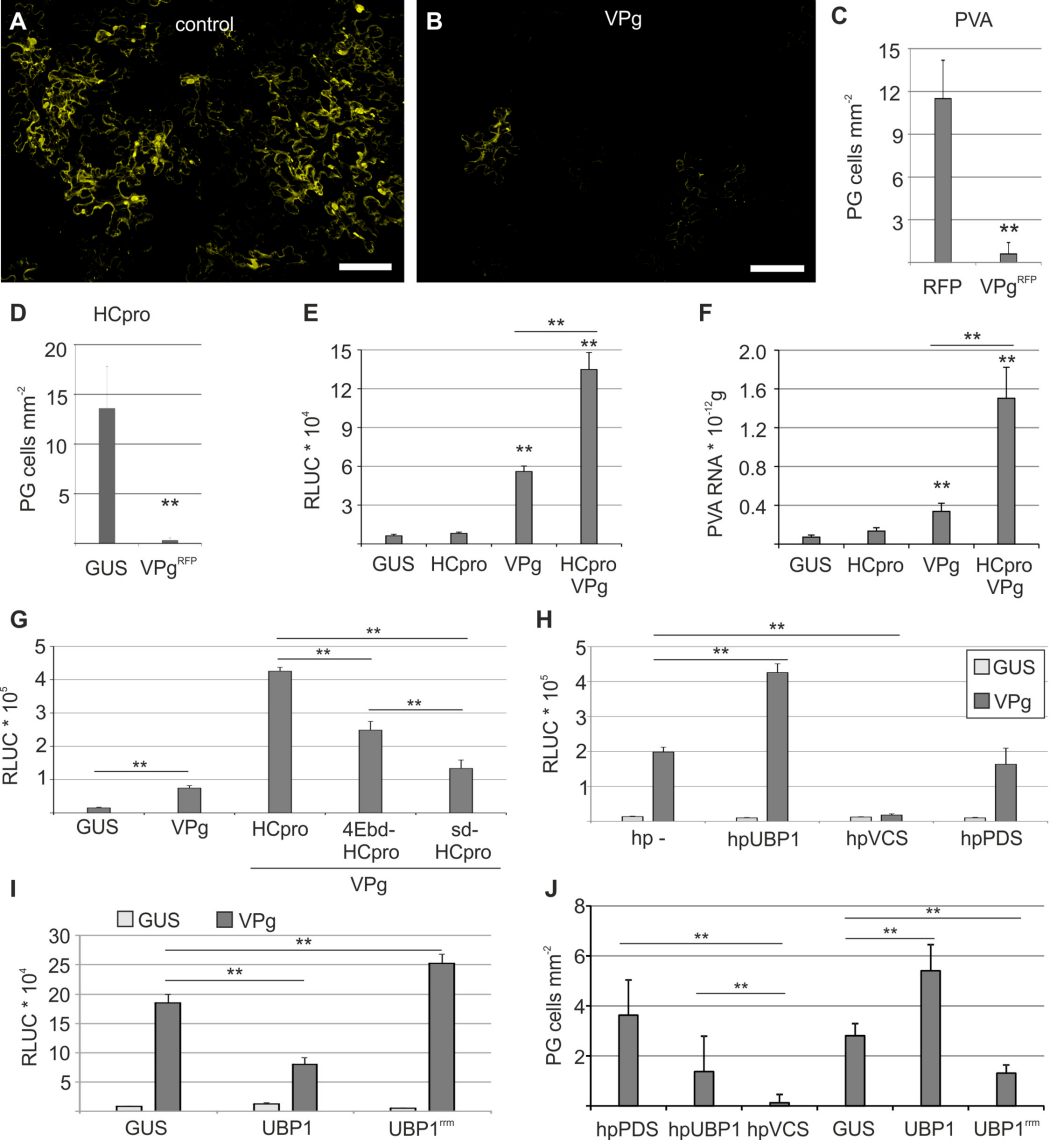
Viral protein VPg together with HCpro and other PG components regulates viral translation. P0^YFP^ and PVA^CPmut^ were expressed to induce PGs in *N*. *benthamiana* leaves, and the effect of co-expressed RFP (A) or VPg^RFP^ (B) on P0^YFP^-labeled PGs was examined by confocal microscopy three days later. RFP fluorescence was used to verify VPg and control expressions. Scale bar; 100 μm. (C—D) Frequency of cells/mm^2^ containing P0^YFP^-labeled PGs in leaf tissues during expression of either RFP or VPg^RFP^. PVA^CPmut^ served as an inducer of the PGs in (C) and HCpro in (D). (E—F) PVA^ΔGDD^ RLUC activities and RNA levels were determined during co-expression of the vRNA with GUS, HCpro, VPg, VPg and HCpro in *N*. *benthamiana* leaves at 3 DAI. (G) PVA^ΔHCΔGDD^ RLUC activity levels were determined during co-expression of vRNA with GUS, VPg, VPg and either HCpro, 4Ebd-HCpro or sd-HCpro in *N*. *benthamiana* leaves at 3 DAI. (H) The capacity of VPg to elevate PVA^ΔGDD^ translation was analyzed during RNA hairpin (hp)-induced silencing of UBP1 and VCS in *N*. *benthamiana* leaves. Empty non-recombined silencing vector (hp-) and phytoene desaturase (PDS) hp-constructs were used as controls. VPg or GUS was expressed with PVA^ΔGDD^ in the different silencing backgrounds and RLUC activity was determined as a reporter of viral protein expression at 5 DAI. (I) The capacity of UBP1 and UBP1^rrm^ to affect VPg promoted PVA^ΔGDD^ RLUC translation was analyzed. Here, GUS or VPg were co-expressed with PVA^ΔGDD^ during ectopic expression of GUS, UBP1, or UBP1^rrm^ mutant lacking RNA-binding domains. All quantitative data is presented as mean and the error bar indicates the standard deviation. (J) The effect of UBP1 and VCS silencing and the effect of UBP1^rrm^ overexpression on PVA-induced PG formation was analyzed by determining the frequency of cells/mm^2^ showing PGs in *N*. *benthamiana* leaves at 3 DAI. (p < 0.01 **, p < 0.05 *).

We have previously linked the PG components P0 and eIF(iso)4E to a virus-specific translational pathway activated by the viral protein VPg [14,15]. Neither P0 nor eIF(iso)4E alone promote PVA RNA expression, but together with VPg they synergistically increase both vRNA and protein levels [15]. Given the central role of VPg in this phenomenon, we set out to analyze a possible link between PVA VPg and PGs. PGs were induced with PVA and visualized with P0^YFP^ that was co-expressed with either RFP-fused VPg^RFP^ or non-fused control RFP. Cells with PGs were uniformly present in the control (Fig 5A), whereas expression of VPg^RFP^ drastically reduced the number of cells with PGs (Fig 5B and 5C). The number of cells containing HCpro-induced granules was similarly reduced upon VPg co-expression (Fig 5D). These data imply that VPg counteracts the existence of PGs.

Next, we analyzed whether HCpro has a role in VPg-activated PVA RNA expression. PVA^ΔGDD^ was co-expressed either with GUS (control), HCpro, VPg or VPg and HCpro together. Ectopic HCpro expression alone had no significant effect on either PVA^ΔGDD^-derived RLUC activity or PVA^ΔGDD^ RNA accumulation whereas co-expression of VPg and HCpro caused their synergistic increase (Fig 5E and 5F). This phenomenon was specific for PVA gene expression since the amount of control *FLUC* mRNA and FLUC activity were unaltered in the same experiment (S5A and S5B Fig). Furthermore, VPg-activated expression of PVA^ΔHCΔGDD^ was increased by providing native HCpro *in trans*, to a lesser extent by 4Ebd-HCpro, and hardly complemented at all by sd-HCpro (Fig 5G). This showed that the function of HCpro in VPg-activated viral RNA expression is reduced by the same mutations that also compromise silencing suppression activity and PG formation.

To gain further insight into how PG components and VPg-activated PVA RNA expression are linked we silenced UBP1, VCS and phytoene desaturase (PDS) as a control, and analyzed VPg- activated PVA^ΔGDD^ expression in these backgrounds. VPg promoted viral RLUC production more in the UBP1-silenced background than in the control background (Fig 5H). As an alternative approach to silencing, we ectopically expressed a UBP1 mutant (UBP1^rrm^), which lacks functional RNA-binding domains and is a dominant-negative suppressor of SG assembly [2]. We found that ectopic expression of UBP1 decreased and UBP1^rrm^ increased VPg-activated PVA expression (Fig 5I), which is in line with the result of the UBP1 silencing experiment. Again, control FLUC production was not affected under these conditions (S5C and S5D Fig). VCS is a component of plant PBs [3,17]. Similarly to P0 silencing [15], VCS silencing completely abolished VPg- activated PVA expression (Fig 5H). The effects of UBP1 and VCS silencing and ectopic UBP1 and UBP1^rrm^ expression on PVA expression became apparent only in the presence of *in trans* expressed VPg suggesting that VPg concentration is a limiting factor for PVA protein production. Successful silencing of VCS and UBP1 was verified by qPCR analysis (S5E Fig). Additionally we observed that PG formation was reduced during UBP1 and VCS silencing, and increased during UBP1 but reduced during UBP1^rrm^ over-expression (Fig 5J) suggesting that translation and PG formation interrelate.

SGs and PBs are dynamically connected to translation in yeast and mammals, and can be either repressed or induced by the translational inhibitors cycloheximide and puromycin, respectively [1]. However, when we isolated PG-containing protoplasts from plant leaves and incubated them with different inhibitors, we could not detect any obvious effects on PGs (S5F–S5H Fig). The insensitivity to translational inhibitors corroborates that PGs do not follow similar dynamics with translation as observed for canonical PBs or SGs.

We conclude that VPg-activated PVA protein production is synergistically increased by HCpro and co-regulated by VCS and UBP1, as well as P0 and eIF(iso)4E [15]. This finding, together with the demonstration that PGs are not present in cells containing a high VPg concentration and that their amount is inversely related to translational regulation, strongly suggests that the formation of PGs is in a delicate balance with VPg-activated PVA protein expression.

### VPg-activated viral translation and PVA infection follow similar pattern of complementation by viral suppressors of RNA silencing (VSRs)

**Fig 6:**
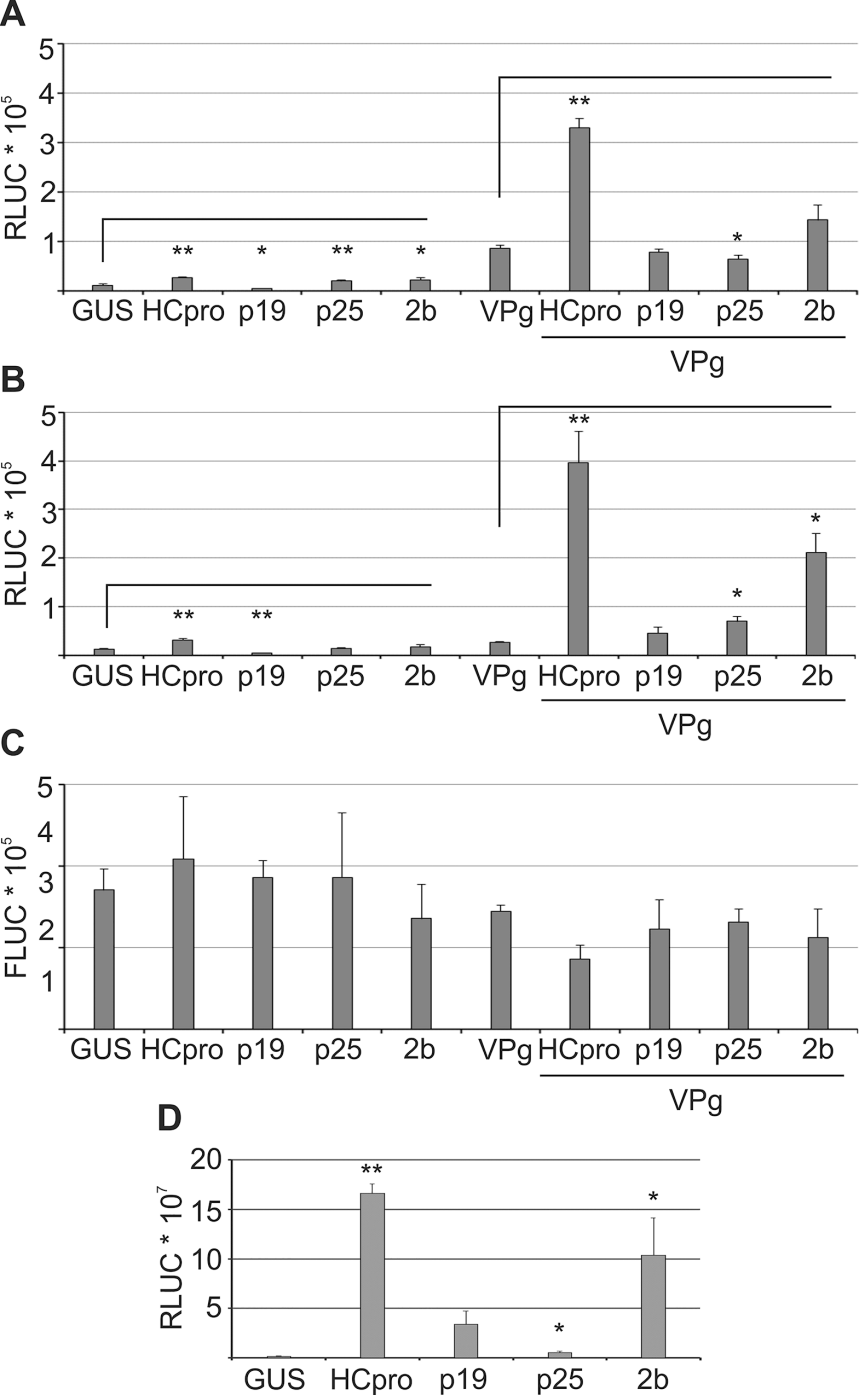
Viral suppressors of RNA silencing HCpro and 2b support VPg-mediated viral translation. Various VSRs were expressed with PVA RNA either alone or together with VPg in *N*. *benthamiana* leaves, and their effect on RLUC activity derived from PVA^ΔGDD^ (A) and PVA^ΔHCΔGDD^ (B) was measured at 3 DAI. In (C), the FLUC activities derived from a non-viral control *FLUC* mRNA co-infiltrated with the PVA constructs was determined from the same samples as in (A and B). FLUC activities were not affected by VSRs and were in average decreased in the presence of VPg showing that RLUC changes in (A and B) were virus-specific. (D) PVA^ΔHC^ was inoculated using a low *Agrobacterium* density (OD_600_ 0.0005) together with indicated viral suppressors of silencing. Potentially successful infections were allowed to spread from the initially transformed cells and viral RLUC activities were determined 6 DAI. Luciferase activities are presented as means and the error bars indicate the standard deviation. (p < 0.01 **, p < 0.05 *).

We analyzed further if other VSRs provided *in trans* could functionally complement HCpro in VPg- activated viral gene expression. Either PVA^ΔGDD^ or its HCpro-lacking derivative PVA^ΔHCΔGDD^ was co-expressed with VPg and various VSRs. PVA^ΔGDD^-derived RLUC activity (Fig 6A) increased considerably more by VPg than that derived from PVA^ΔHCΔGDD^ (Fig 6B), the difference being a 7.8 fold versus a 2.2 fold increase respectively, supporting that HCpro can synergistically increase VPg-activated PVA protein production as implied also in Fig 5E and 5G. When HCpro was expressed with PVA^ΔHCΔGDD^, VPg increased its expression as strongly as that of PVA^ΔGDD^ showing that this function of HCpro can be efficiently complemented *in trans*. Of the other VSRs, 2b complemented the absence of endogenous HCpro moderately and P19 and P25 weakly. When control FLUC activity was analyzed similarly, it remained essentially unaltered (Fig 6C). These results showed that HCpro together with VPg promote viral gene expression and, that the non-related VSR 2b can functionally complement HCpro in this process to some extent.

We also analyzed the capacity of other VSRs to complement HCpro during PVA infection. For this purpose, HCpro was deleted from PVA^WT^ (S1 Fig) and expressed together with different VSRs whereas no ectopic VPg expression was applied here. Different degrees of complementation were observed depending on the VSR (Fig 6D). The low RLUC activity from the PVA^ΔHC^ in the GUS control samples verified an impaired infection. HCpro supplied *in trans* complemented efficiently the lack of endogenous HCpro. Of the other VSRs, 2b was most efficient in complementing PVA^ΔHC^ expression. Substantial complementation was observed also by P19 whereas P25 was weak in this function. The level of complementation in PVA infection was comparable to the one observed for VPg-activated viral translation (compare Fig 6A and 6D), supporting the importance of the VPg-driven PVA gene expression mechanism during authentic infection.

### PG components are important for PVA infection

**Fig 7:**
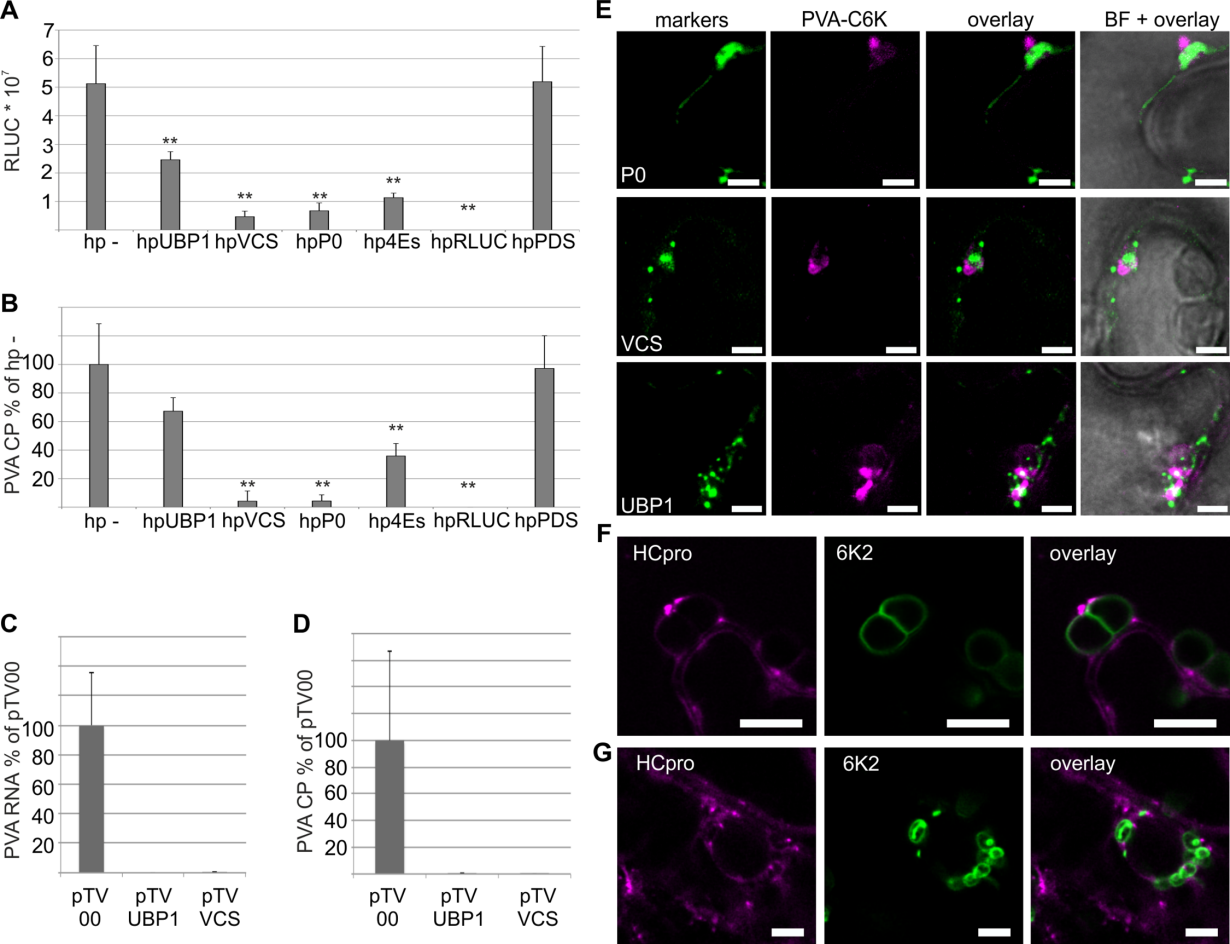
PGs are important for PVA infection and they can associate with viral replication complexes. (A-B) PG-associated proteins UBP1, VCS, P0 and eIF4E/(iso)4E were silenced in *N*. *benthamiana* leaves via hairpin (hp)-constructs. Empty hairpin construct (hp-) and hpPDS were used as controls and hpRLUC to block infection via vRNA silencing. To initiate PVA infection from individual cells, RLUC-tagged PVA^WT^ was inoculated using a low *Agrobacterium* density (OD_600_ 0.0005). Infection was then allowed to spread from the initially infected cells in the silencing backgrounds. Six days later, viral RLUC activities (A) and the coat protein levels (B) were determined and they are presented as means with error bars indicating the standard deviation. (C—D) PG-associated proteins UBP1 and VCS were silenced in *N*. *benthamiana* using TRV-mediated VIGS followed by mechanical inoculation of PVA and quantification of PVA in systemically infected leaves using ELISA (C) and qPCR (D) at 7 DAI. (E) Plants were inoculated with PVA tagged with an additional copy of 6K2^CFP^ and infection was let to spread into systemic, non-inoculated leaves. P0^YFP^, VCS^YFP^ and UBP1^YFP^ were subsequently expressed in the systemically infected leaves using *Agrobacterium* infiltration and imaged together with 6K2-labeled VRCs. The overlay shows PG-markers and VRCs as separate structures adjacent to each other in the presented single-layer images. Scale bar; 3 μm. (F—G) *N*. *benthamiana* plants were inoculated with PVA-HC^RFP^-6KY icDNA tagged simultaneously with HCpro^RFP^ and an additional copy of 6K2^YFP^ (see schematic presentation of the construct in S1 Fig) using *Agrobacterium* infiltration (F) and virion inoculation (G). PGs were visualized via HCpro^RFP^ and VRCs via 6K2^YFP^ in upper, non-inoculated leaves by confocal microscopy upon development of systemic infection, and presented as single layer images. Similar to (E) PGs were detected in the vicinity of VRCs as well as separated from them. Scale bar; 10 μm (upper) and 5 μm (lower). (p < 0.01 **, p < 0.05 *).

The role of plant RNA granule proteins P0, VCS, UBP1, eIF(iso)4E, all of which localize together with HCpro in PGs (see Figs 2 and 7 and S3) and regulate VPg-activated PVA gene expression (Fig 5 and [15]), was further studied during PVA infection by silencing them with RNA hairpin inducers. We have already earlier reported that P0 and eIF4E/eIF(iso)4E silencing reduces PVA infection [15]. Silencing of VCS and UBP1 was verified by RT-qPCR (S5E Fig). Three different controls were used: hpRLUC to target the viral genome, hpPDS to target a non-related mRNA and an empty hairpin (hp-) vector. We infiltrated *Agrobacterium* carrying the RLUC-tagged PVA^WT^ at a very low concentration (OD_600_ 0.0005) to initiate infection from a few cells only and thereby to allow infection to spread via cell-to-cell movement in the inoculated tissue. When viral RLUC activity and viral CP concentrations were determined at 6 DAI, we found that silencing of all PG components, but especially VCS and P0, reduced RLUC and CP accumulation (Fig 7A and 7B). Next we used GFP-tagged PVA to analyze if viral cell-to-cell movement was affected in the backgrounds deficient in PG components. Both the number of infection foci per leaf area and the speed of cell-to-cell spread of infection remained essentially unaltered during silencing of PG components (S6 Fig). In agreement with reduced PVA RLUC activity levels (see Fig 7A), the intensity of PVA-derived GFP was low throughout the whole tissue silenced for PG-components. To visualize viral spread, the images in S6 Fig were acquired with increased sensitivity and are not comparable with each other in terms of fluorescence intensity.

In order to replicate these results in another experimental context, we used TRV-mediated virus-induced gene silencing (VIGS) to down-regulate VCS and UBP1 in *N*. *benthamiana* (S6E Fig). 10 days after initiation of VIGS, lower leaves were mechanically inoculated with PVA, and accumulation of virus was analyzed seven days later in systemically infected leaves by ELISA and qPCR (Fig 7C and 7D). Both ELISA and qPCR showed that PVA levels were severely reduced in both VCS and UBP1 VIGS plants, residing barely above the detection limit. These results showed that PG components UBP1 and VCS are important to achieve high virus titer at single cell level and systemically, although both the efficiency of infection initiation and cell-to-cell spread in locally inoculated leaves was unaltered in UBP1 and VCS deficient backgrounds.

### PGs exist both separated and in association with viral replication complexes

Very little is known about how potyviral RNA produced within and released from viral replication complex (VRC) integrates with the host mRNA pathways to be directed either to translation or degradation. Our hypothesis was that PGs could represent a structure required between replication and translation to protect against degradation of vRNA and therefore they could associate closely with VRCs. In order to visualize VRCs we engineered PVA to express the VRC marker protein 6K2 fused to CFP (S1 Fig). It has been shown that the presence of dsRNA coincide with fluorescence emitted by 6K2-GFP in TuMV-infected cells [24], proving that tagged 6K2 constitutes a good marker for the visualization of potyviral VRCs. Virus infection was allowed to spread into systemic non-inoculated leaves and these leaves were then infiltrated with *Agrobacterium* carrying the expression constructs for P0^YFP^, VCS^YFP^ or UBP1^YFP^. Imaging of these leaves 3 days later revealed that VRCs marked by 6K2 were distinct from the granule structures labeled by P0^YFP^, VCS^YFP^ and UBP1^YFP^ (Fig 7E). As hypothesized, P0-, VCS- and UBP1-marked structures were found adjacent to VRCs, but also apart from them.

Finally, we engineered PVA to express HCpro^RFP^ and an extra copy of YFP-tagged 6K2 from its genome (S1 Fig). With this construct we aimed at visualizing PGs under circumstances as closely mimicking those during natural infection as possible. This recombinant virus, PVA-HCpro^RFP^-6KY, retained its infectivity and was able to spread systematically regardless whether the infection was initiated by *Agrobacterium* infiltration (Fig 7F) or mechanical inoculation of PVA-HCpro^RFP^-6KY particles (Fig 7G). When we visualized the spatial association of VRCs and PGs in the cells of systemically infected leaves, HCpro granules were distinct from potential VRCs labeled by the 6K2 marker (Fig 7F and 7G). The PGs were smaller under these close to natural conditions when compared to those detected during ectopic expression of fluorescent granule protein fusions (see Figs 1 and 2 and 4 and S2). It is possible that the small size of PGs in the presence of PVA^WT^ may have led to an underestimation of the frequency of PG-containing cells in (Figs 1G and S2D) due to the resolution limit of the epifluorescence microscopy. To summarize, these results showed that VRCs and PGs are spatially distinct but often closely associated structures during infection. The ratio between PG inducing (HCpro; Fig 1) and repressing (VPg; Fig 5) viral components, and viral processes such as particle assembly and RNA silencing likely contribute to the overall size and quantity of the observed PGs during natural infection.

## Discussion

**Fig 8:**
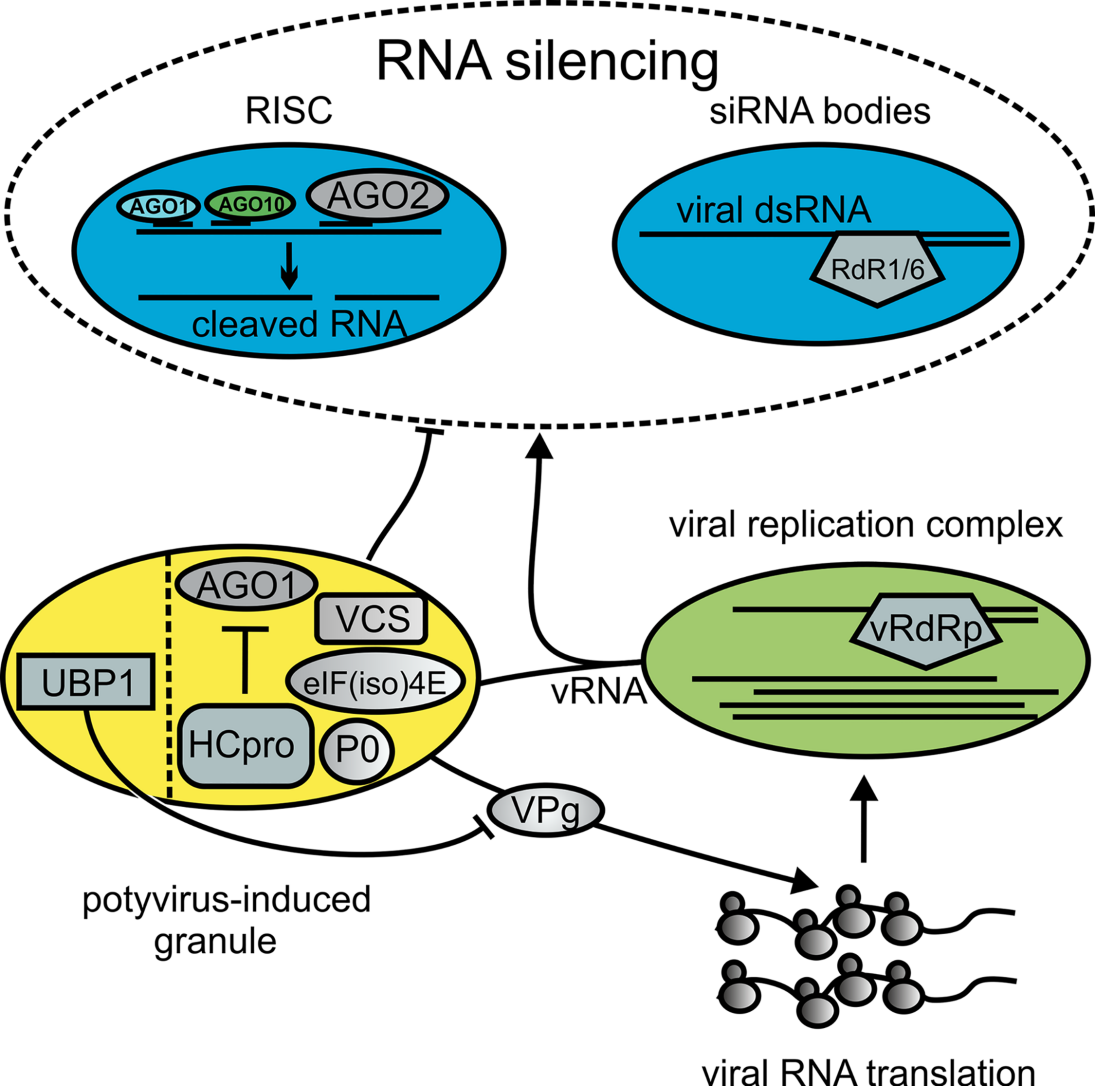
A model for PGs in PVA infection. Viral RNAs engage in different processes after replication including translation. This involves recruitment of the cytoplasmic translational machinery, which potentially exposes viral RNAs to hostile cytoplasmic conditions. RNA silencing-associated structures, potyvirus-induced granules and polysomes may overlap to a yet unknown extent, but are illustrated here as alternative destinations for replicated viral RNA. HCpro, UBP1, VCS, P0 and AGO1 are redistributed to PGs together with translationally inactive viral RNA when active viral translation is not supported. PG assembly and translational repression are promoted by UBP1 whereas translational activation of viral RNA occurs via an cooperative action orchestrated by VPg together with PG components including P0, VCS, HCpro and eIF(iso)4E. VPg disrupts the formation of PGs, underscoring that PG formation and viral translation are interrelated processes in PVA RNA gene expression. We propose that PGs play an important role in protecting viral RNA from antiviral silencing and thereby necessary to achieve optimal virus accumulation in plants where RNA silencing is active.

In this study we show that PVA infection induces RNA granules referred to as PGs. Knockdown of individual PG components resulted in low PVA infection levels, suggesting that PGs have an important role in PVA accumulation. We found that HCpro is the viral component responsible for PG induction and HCpro localizes abundantly to PGs together with AGO1. Amino acid substitutions impairing the capacity of HCpro to suppress RNA silencing concomitantly abolished PG induction. However, as PVA HCpro mutants could affect these activities by independent ways, this alone doesn't allow us to conclude if PGs participate in HCpro-mediated silencing suppression or other unrelated functions. Mutant TuMV-AS9 impaired in silencing suppression infects silencing-deficient *dcl2 dcl4* plants with similar strength as WT TuMV, but is unable to infect WT Arabidopsis Col-0 plants due to lack of silencing suppression [23]. Interestingly, the mutated HCpro of TuMV-AS9, which is similar to our sd-HCpro, does not induce PGs (Fig 4G). On this basis PGs appear to be important for potyvirus infection only when host silencing is active. A recent report described a mutation in HCpro that increased the formation of cytoplasmic foci, likely PGs. Simultaneously this mutation increased the capacity of HCpro to suppress RNA silencing [25], also lending indirect support to silencing suppression-related PG functions. We suggest that viral translation is coupled to PGs because many PG components affect VPg-activated PVA RNA translation as demonstrated in this study and in our previous studies [14,15]. We speculate that vRNA can enter RNA metabolic pathways involving PGs, translation and RNA silencing rapidly after replication. This emerging picture of the post-replication route of PVA RNA includes that suppression of vRNA silencing occurs in favor of translation in a manner regulated by viral proteins HCpro and VPg and, host proteins including P0, VCS, eIF(iso)4E and UBP1 (Fig 8).

PBs and SGs have essential roles in mRNA metabolism, including mRNA translational regulation, storage, decay and silencing [26]. This area has remained less studied in plants than in yeast or animal cells, but there is growing evidence that plant, yeast and animal PBs and SGs share similar functions [2,3,5,27–29]. Our attempts to identify compositional redundancy of PGs with plant PBs and SGs revealed that PGs contain markers of both granule types. These include VCS, DCP1 and AGO1 of PBs [3,17], and UBP1 and eIF(iso)4E of SGs [2,5]. PBs and SGs have been shown to fuse under certain stress conditions [20,26,30], which suggest that markers commonly distinguishing PBs from SGs can also overlap. The insensitivity of PGs to cycloheximide, actionomycin D and puromycin treatments distinguishes PGs from PBs and SGs, which both are dynamically associated with the changes in the overall translational activity of the cell [1].

We hypothesize that many RNAs, including the PVA RNA, associate with PGs. Animal PBs assemble as a consequence of activated RNA silencing, and knock down of RNA silencing genes, e.g. AGO proteins, results in the absence of PBs [31]. Our results show that P0 containing PGs are abundant during HCpro expression when compared to the VSRs P19, 2b and P25. Some PGs formed in the presence of P19 and the HCpro-less PVA infection was complemented by P19. This is in line with the finding that P19 can complement HCpro-less *Plum pox virus* (PPV; genus Potyvirus) infection [32] as well as silencing suppressor-deficient HCpro mutant of TuMV [23]. Whereas no P0-labeled PGs were found with 2b, it still complemented PVA infection and VPg-driven protein production, and supports that PGs are important for HCpro-mediated suppression of RNA silencing. This may be a function corresponding to 2b-mediated AGO1 inhibition in PB-associated RNA-induced silencing complexes [9,28]. A recent study demonstrated a central antiviral role for AGO2 in TuMV infection, whereas AGO1 seemed to have a less important role [33]. The authors expressed their concern in interpreting infection results for AGO1 due to the large number of genes that are deregulated when AGO1 is disrupted, including AGO2 induction. They also speculated that AGO1 pools may a have limited access to viral siRNAs during TuMV infection, which could be attributable to PG functions. In addition to its slicing function, AGO1 mediates miRNA-directed translation repression *in planta* [34]. Based on our finding that AGO1 localizes to and promotes PG formation, we anticipate that AGO1 has a role in potyvirus infection that is a matter to study further. It is also possible that other AGOs including AGO2 localize to PGs and regulate infection. Taken our findings together, we propose that P0-containing PG induction is not a general outcome of VSR interference with RNA silencing but related to specific properties of HCpro, as most plant viruses encode VSRs without shared sequence homology that suppress RNA silencing by different mechanisms [35]. Other markers need to be applied when assessing more generally the impact of VSRs on RNA granule formation, whereas P0 could be considered as a hallmark protein of HCpro-induced PGs. Also animal virus infections can induce compositionally unique RNA granules [10].

A growing body of evidence suggests interdependence between mRNA quality control and RNA silencing. Genetic screens in *Arabidopsis* have identified several endogenous suppressors of RNA silencing, many of which localize to PBs, where they function in mRNA maturation and decay [8,36–38]. Aberrant RNAs and mRNAs, which become decapped in PBs, can serve as substrates for RdRps to initiate or maintain transgene silencing [8]. PVA VPg has the capacity to suppress RNA silencing, which has been demonstrated in a co-suppression assay [39]. We analyzed the capacity of VPg to suppress RNA-hairpin triggered silencing, but could not detect such an activity (S4C Fig). In contrast to RNA hairpin-induced silencing, co-suppression depends on RdRps to convert single-stranded RNA templates into double-stranded RNAs that go on to trigger silencing [40]. Recently VPg was further shown to interact with the RdR6-silencing body protein SGS3 in favor of PVA infection [41], and RdRp1 and RdRp6 were demonstrated to modulate host defense against potyviruses [23]. We show that VPg, HCpro and many PG components support vRNA expression in a synergistic manner (this study; [15]). The mechanism is specific for vRNA as translation of a control *FLUC* reporter mRNA remains unaffected. One possibility is that both HCpro and VPg promote viral gene expression by targeting different functions of the silencing machinery and by associating with RNA granule proteins, P0, eIF(iso)4E and VSC assisting in efficient viral translation (this study; [15]). UBP1 repressed VPg-activated gene expression, which is in agreement with the fact that plant UBP1 is mainly associated with translationally silent mRNAs [5]. UBP1 contains a prion-domain and several RNA-binding domains, which are essential in driving SG aggregation [2], and accordingly UBP1 also promoted PG aggregation. The positive role of PGs would be sufficient to explain the important role of UBP1 in promoting PVA infection despite its repressive impact on PVA translation. Considering further that both P0 and VCS are involved in PG assembly and promote VPg-activated PVA RNA expression, these processes appear to be intimately coupled.

Plus-strand RNA viruses replicate their RNA genomes within membranous viral replication complexes with the aid of many viral and host proteins [42]. The replication process includes the production of mRNA-like RNAs for translation. Numerous RNA binding proteins and their co-factors control canonical host mRNAs from transcription to decay. The mechanisms of targeting vRNAs to translation from cytoplasmic replication complexes, possibly utilizing the extensive cellular mRNA regulatory network, have remained largely enigmatic. We consider the possibility that canonical RNA granule components associate with PVA RNA upon exit from replication complexes as we observed PGs frequently in close vicinity of potential replication sites. Targeting of AGO1 to these foci suggests that this RNA silencing protein is potentially competing for the released vRNA but is suppressed in PGs by HCpro. This lends indirect support from our notion that 2b can substitute HCpro with high efficiency in viral translation and infection, as 2b was demonstrated to suppress AGO1 in PBs [28]. Upon successful suppression of RNA silencing by HCpro, VPg can further promote viral translation together with P0, VCS and eIF4E/eIF(iso)4E. Coupling of translation and replication has been proposed for potyviruses [43–45], as well as for several other plus-stranded RNA viruses [46]. When considering that PG components are essential for efficient PVA RNA translation and strong infection, replication-coupled translation involving PGs appears plausible, and the frequent association of putative VRCs and PGs support this idea.

Taken together, we have demonstrated that potyvirus infection induces formation of RNA granules, PGs, which are neither canonical PBs nor SGs. Furthermore, they are not VRCs although PGs can spatially associate with them. We show that PGs are RNA granules harboring the silencing suppressor HCpro. PG assembly is promoted by several cellular RNA granule components, as shown for UBP1, P0 and AGO1, and involves their simultaneous redistribution to these structures. We propose that HCpro functions related to regulation of viral gene expression after replication involve PG assembly, possibly altered AGO1 activity achieved by its co-assembly into PGs, and its role in VPg-activated translation. The synergism between HCpro and VPg in promoting viral translation may account for the silencing suppression pathway as well. While many outstanding questions about the molecular mechanisms associated with these events remain to be studied, this study reveals that formation of PGs and viral translation are interrelated processes, which may also couple with RNA silencing suppression, and the essential role of these pathways in PVA accumulation.

## Materials and Methods

### Plants and antibodies

*Nicotiana benthamiana* plants were grown in a greenhouse at 22°C for an 18 h day and at 18°C for a 6 h night and used for experiments at the 4- to 6-leaf stage. All plant experiments were conducted on *N*. *benthamiana*. YFP was detected with anti-GFP antibody (Santa Cruz; SC-9996), RFP with anti-RFP (SignalChem; R46-61M-100) and secondary antibody was HRP-conjugated anti-mouse (Promega; W4021). HCpro was detected with polyclonal PVA HCpro antibody produced in rabbit and AGO1 with polyclonal *Arabidopsis thaliana* AGO1 antibody (Agrisera; AS09 527) produced in rabbit. Secondary antibody was HRP-conjugated anti-rabbit (Promega; W4011). P proteins were detected with human autoimmune disease serum against ribosomal P antigen (Immunovision; HP0-0100) and secondary antibody was HRP-conjugated goat anti-human antibody (Jackson ImmunoResearch; 109-001-003).

### Construction of recombinant viruses

PVA^WT^, PVA^CPmut^ and PVA^ΔGDD^ have been described [16]. PVA-6KY: A gateway compatible 6K2 cistron was produced by PCR from the PVA genome and recombined via pDONR/Zeo into pGWB41 to generate a 6K2-YFP fusion. A 6K2-YFP fragment was then generated by PCR and cloned in between of NIb and CP genes in the PVA icDNA as described for GFP previously [47]. PVA-C6K: With an analogous approach taken for PVA-6KY, 6K2 was recombined into pSITEII-2C1 to generate a CFP-6K2 fusion, which was amplified by PCR and cloned in between NIb and CP into the icDNA of PVA. PVA-HC^RFP^-6KY: The HCpro cistron was fused to a Strep-III-tag and TagRFP in the following order: Strep-III-TagRFP-HCpro. The recombinant HCpro was then used to replace the native HCpro in PVA-6KY icDNA to generate PVA-HC^RFP^-6KY. PVA^ΔHC^: HCpro was deleted by outward PCR of a SexAI-NruI fragment containing P1 C-terminus-HCpro-P3 N-terminus in pGEM-T Easy, leaving the 3 first HCpro codons in order to retain the processing site for P1 intact when fused to P3. The P1-P3 fusion fragment was re-inserted to PVA icDNA using SexAI and NruI restriction sites replacing the HCpro containing fragment in the PVA genome. All resulting recombinant PVA icDNAs were transferred from pUC18 to the binary vector pRD400 for *Agrobacterium*-mediated transformation.

For RNA imaging, the RLUC-tagged PVA^∆GDD^ icDNA was cloned into pDONR/Zeo and further into the gateway-compatible binary vector that adds 16 bacteriophage λ B-box elements to the 3´ end as described in [19].

The PVA HCpro cistron was amplified with gateway-compatible primers and recombined into pDONR/Zeo for mutagenesis. The silencing deficient HCpro mutant (sd-HCpro) was produced by site-directed mutagenesis converting R237, R238, R245, and K246 to alanines, reported to abolish silencing suppression activity for TEV HCpro [21]. The eIF4E binding deficient HCpro mutant (4Ebd-HCpro) was produced by mutating Y344 and L349 to alanines. These changes were reported to decrease the affinity between eIF(iso)4E and PVA HCpro in [22]. TuMV HCpro and the AS9 mutant HCpro cistrons were amplified by PCR using pCB-TuMV-GFP and pCB-TuMV-AS9-GFP plasmid DNAs [23] as template. The PCR products were cloned into pENTRY-TOPO and recombined into pGWB442 to obtain N-terminal YFP-fusions.

### Plant expression constructs

The generation of P0 and eIF(iso)4E gateway-compatible cDNAs is described in [15]. Gateway-compatible cDNAs of UBP1 and UBP1^rrm^ were generated by PCR using plasmids described in [2] as templates. Gateway-compatible cDNAs of AGO1, P1 and P2 were amplified from a *N*. *benthamiana* cDNA library followed by recombination into pDONR/Zeo prior to final recombination to plant expression vectors. P0 was recombined into pGWB41 to generate a C-terminal YFP-fusion protein, P1 and P2 into pGWB42 giving N-terminal YFP-fusions, AGO1 into pSITEII-2C1 for N-terminal CFP-fusion, UBP1 and UBP1^rrm^ into pGWB18 for 4X myc-fusions, UBP1 into pGWB42 and pGWB45 for YFP- and CFP-fusions, VPg into pSITE-4N1 mRFP fusion, HCpro (including 4Ebd- and sd-HCpro), eIF(iso)4E into pSITEII-6C1 for TagRFP-fusion. pGWB vectors are described in [48] and pSITE vectors in [49]. Non-fused YFP was expressed from pGWB42 and RFP from pSITE-4C1. The constructs to express GUS, VPg, P0, eIF(iso)4E, and PVAs are described in [14,15]. For the silencing of UBP1, VCS and PDS, gateway compatible cDNA fragments were generated from an *N*. *benthamiana* cDNA library and recombined via pDONR/Zeo into pHELLSGATE8 (pHG8) [50], similarly to P0 [15] and eIF4Es [14] (see accession numbers for sequences below). All plant expression cassettes were transformed into *Agrobacterium tumefaciens* strain C58C1 pGV2260 and their expression was initiated by *Agrobacterium* infiltration as specified in connection to each experiment. Expression constructs for DCP1 and VCS fluorescent protein fusions are described in [3].

### Confocal microscopy

Fluorescent proteins were commonly imaged in *N*. *benthamiana* leaves 3 days after *Agrobacterium*-mediated transformation unless otherwise specified. Confocal laser scanning microscopy was performed on a Leica TCS SP5II confocal microscope. When YFP was imaged alone or together with CFP, excitation was performed with an argon laser at 514 nm and emission recorded at 525–555 nm (DD 458/514 beam splitter). CFP was excited with the argon laser at 458 nm and emission recorded at 470–500 nm. When YFP was co-imaged with RFP, excitation was performed with the argon laser at 488 nm and emission recorded at 525–555 nm. RFP was excited with DPSS 561 nm laser and emission recorded at 570–620 nm (DD 488/561 beam splitter). The sequential scanning mode was applied for co-imaging of different fluorescent proteins. All imaging was conducted using live plant leaf tissue mounted between a cover and objective glass and immerged in water. Tissue images were acquired using a 20X objective and represent Z-stacks taken at 1μm intervals. Single cell images were acquired using a 63X objective representing either Z-stacks taken at 500 nm intervals or single layer images as indicated. All images were deconvoluted using Autoquant X3 software and the presented images were obtained using Imaris software applications.

### PVA inoculation and quantification

*Agrobacterium* infiltration was used as a method to initiate both PVA RNA and ectopic expression of viral and host proteins. Infiltration of *Agrobacterium* carrying various PVA icDNAs, and quantification of viral RLUC activity and viral coat protein by ELISA was as described in [16]. Briefly, *Agrobacterium* carrying PVA was infiltrated as specified into a minimum of two parallel plants for each condition. When specifically mentioned PVA infection was initiated by mechanical inoculation. For mechanical inoculation systemically infected *N*. *benthamiana* leaves were harvested at 10 dpi and homogenized in TBS buffer (150 mM NaCl, 50 mM Tris-HCl, pH 7.5) with the ratio of 1 g of leaves per 5 ml of buffer. The infected tissue homogenate was used to inoculate *N*. *benthamiana* plants using carborundum as abrasive. Systemically infected leaves were analyzed either by confocal microscopy, ELISA or qPCR at 7 DAI.

For qPCR, total RNA was extracted using TRIZOL reagent according to the manufacturer’s recommendations. 1 μg of total RNA was treated with Thermo Scientific RQ1 DNAse at 37°C for 30 min and further used for cDNA synthesis using Thermo Scientific RevertAid H Minus First Strand cDNA Synthesis Kit following the manufacturer’s recommendations. qPCR was carried out using 2x Maxima SYBR Green qPCR MasterMix according to the manufacturer’s recommendations. The quantitative data comprise four replicate samples for each condition, and error bars report their standard deviations. Student’s t-test was applied to calculate the significance of the differences between the samples.

### Gene-silencing by transient expression of hairpin RNA and VIGS

Gene silencing was achieved using *Agrobacterium*-mediated expression of RNA hairpins from the pHG8 vector containing gene-specific inserts. Translation and infection assays in the silenced backgrounds with PVA and possible co-expressed proteins were done by co-infiltration with pHG8 constructs as specified in the text and figure legends. The level of gene silencing for UBP1 and VCS was analyzed using RT-qPCR four days after infiltrating *Agrobacterium* expressing pHG8::UBP1, pHG8::VCS and empty pHG8 (control). Silencing of P0 and eIF4E/eIF(iso)4E using pHG8 constructs was demonstrated previously [14,15].

For VIGS, the gateway compatible fragments of VCS and UBP1 in pDONR/Zeo were recombined into the gateway-compatible TRV2 vector described in [51]. These gene-specific fragments were the same that was used in constructing the pHG8 silencing constructs. Empty TRV2, and TRV1, were those described in [52]. VIGS was initiated according to standard procedure followed by mechanical inoculation of PVA 10 days later. Upper, non-inoculated leaves were harvested seven days later for the analysis of PVA accumulation and estimation of VCS and UBP1 silencing levels.

### Quantification of PGs

*N*. *benthamiana* leaves were infiltrated with *Agrobacterium* to express proteins as specified, and the amount of cells showing PGs was scored by epifluorescence microscopy (Zeiss Axio Scope.A1 microscope) with appropriate filters for GFP and RFP three days later unless otherwise stated. Cells were counted in the epidermal tissue using a viewing area of ~ 4.6 mm^2^, which contains roughly 200 cells. To facilitate quantification of cells showing PGs and differences between conditions, the densities (OD_600_) of infiltrated *Agrobacteria* was adjusted low enough to have a non-saturated response, i.e. aiming at approximately 1 out of 10 to 50 cells showing PGs. The OD_600_ used was 0.1 for P0^YFP^, PVAs and HCpro^RFP^ unless otherwise stated. Using higher densities of the strong PG-inducers HCpro or PVA^CPmut^, PGs were present in practically all cells. Cells showing PGs were counted and are given as cells per mm^2^. For each condition, three different plants were used, and from each plant, the number of cells with PGs was counted from three separate leaf areas (n = 9).

### Isolation of PGs

Leaves were infiltrated with P0^YFP^ and PVA using *Agrobacterium* OD_600_ 0.2. At 3 days after infiltration, leaves were homogenized in 3 ml buffer (100 mM Tris-HCl pH 8, 150 mM NaCl, 5% sucrose, protease inhibitor cocktail from Roche) g^-1^ of leaf. After filtration through a damp coffee filter, the lysate was cleared by centrifugation 2 X 5 min 500 x *g* at + 4°C, loaded on a 40% sucrose cushion above a 80% sucrose cushion and centrifuged 40 min 3000 x *g* at + 4°C. The 40 to 80% sucrose interface was collected and diluted 5-fold with buffer supplemented with 0.5% NP-40, loaded on an 80% sucrose cushion and centrifuged for 40 min 3000 x g at + 4°C. The 80% sucrose cushion and the interface were collected representing the PG-fraction. For RNAse A treatment, PG fractions were diluted 5-fold with buffer and centrifuged into a pellet (10 min 3000 x g + 4°C), re-suspended in buffer, followed by incubation with 1 μg μl^-1^ RNAse A for 3 h at room temperature. Centrifugation for 5 min 3000 x *g* was used to separate RNAse A or control treated PG fractions into soluble and pellet fractions, followed by analysis using a fluorescence plate reader (Fluoroskan Ascent FL; Thermo Scientific). Relative fluorescence was obtained by plotting the values onto a serial dilution standard derived from a dense PG fraction. For strep-affinity purification, appropriate PG fractions were supplemented with 100 μg ml^-1^ avidin (IBA) and incubated rotating for 1 h at + 4°C with a strep-tactin macroprep matrix (IBA), washed 2 X 2 ml buffer and eluted with 2.5 mM desthiobiotin (IBA). The elutes were concentrated in a microcon YM-10 (Millipore), the RNA extracted with total RNA extraction kit including DNAse I treatment (Qiagen), and Superscript III (Invitrogen) was used for RT. Detection of viral RNA was achieved by PCR using the same primers as in RT-qPCR of PVA RNA [14].

### Isolation of protoplasts with PGs

Leaves of *N*. *benthamiana* were agroinfiltrated to express P0^YFP^ and PVA^CPmut^ in order to induce PGs. Two days after infiltration, leaves were sliced and incubated overnight with cellulase and maserozyme, and protoplasts were harvested, mixed into 50% percoll followed by percoll-based density fractionation in 0/20/50% gradient. Protoplasts on top of the 20% Percoll were collected and subsequently incubated in the presence of 10μg ml^-1^ puromycin (Sigma P8833), cycloheximide (Sigma C6679) or actinomycin D (Sigma A1410) for three hours and imaged using confocal microscopy.

### Accession numbers

NM111960 (*A*. *thaliana* P0), FN666434 (*N*. *tabacum* eIFiso4E), NP_171618 (*A*. *thaliana* P1), NP_180340 (*A*. *thaliana* P2), CAB75429 (*N*. *plumbaginifolia* UBP1), KC790391 (*N*. *benthamiana* UBP1 silencing fragment) and KF218187 (*N*. *benthamiana* VCS silencing fragment).

## Supporting Information

S1 Fig Modified PVA infectious cDNA constructs used in this study. *Renilla* luciferase (*RLUC*) inserted into the PVA icDNA between RNA-dependent RNA polymerase (NIb) and coat protein (CP) genes [16]. PVA^CPmut^ and PVA^ΔGDD^ represent movement- and replication-deficient *Rluc*-tagged PVAs, respectively (mutated amino acids indicated) [16]. PVA-6KY and PVA-C6K are PVAs modified with an extra copy of 6K2-YFP or Cerulean FP-6K2 gene, respectively. PVA^ΔHC^ is PVA virus construct with a deleted HCpro gene. PVA^ΔHCΔGDD^ is PVA^ΔGDD^ having HCpro gene deleted and PVA^B-box^ has B-box RNA element fused to the 3’UTR of PVA^ΔGDD^. PVA-HC^RFP^-6KY has HCpro fused to RFP and StrepIII-tag in addition to the extra 6K2-YFP.
(TIF)

S2 Fig P0^YFP^ accumulate and form PGs in the presence of PVA, related to Fig 1. (A) GUS (control) and P0^YFP^ were co-expressed with *RLUC*-tagged PVA^WT^ and viral RLUC activity was analyzed at 7 days after infection (DAI). To mimic natural infection process PVA was inoculated using a low *Agrobacterium* density (OD_600_ 0.0005) to initiate infection from single cells from which the infection then spreads via cell-to-cell movement (as in S6 Fig). RLUC activity reporting for the activity of viral gene expression is presented as mean + standard deviation and a student’s t-test shows that viral RLUC activity is significantly enhanced in P0^YFP^ samples when compared to the control samples (p < 0.05 *), showing that P0^YFP^ promotes PVA gene expression similarly to non-fused P0 [15]. This validated the use of fusion protein P0^YFP^ in studying the role of P0 in PVA infection. (B) P0, YFP and P0^YFP^ were expressed in the presence and absence of PVA^WT^. YFP and P0^YFP^ accumulation was detected by anti-GFP western blot analysis. The non-tagged P0 served as a negative control for YFP detection. P0^YFP^, but not YFP, accumulation was enhanced in the presence of PVA, suggesting that PVA does not cause accumulation of any co-expressed protein but rather it is P0 specific response. The membrane was also stained for total protein using Ponceau S to verify loading accuracy and is shown at the position of the large RuBisCO subunit. (C) P0^YFP^ expression was analyzed, in parallel with confocal microscopy imaging in (D), by western blotting using anti-GFP antibody. The membrane was also stained for total protein using Ponceau S to verify loading and is shown at the position of the large RuBisCO subunit. Correlating with PG-formation in (D), P0^YFP^ accumulation was enhanced in the presence of all PVA variants compared to the non-infected mock sample. (D) P0^YFP^ was expressed together with PVA^WT^, PVA^CPmut^, PVA^∆GDD^ or GUS (mock) using *Agrobacterium*-mediated transformation of *N*. *benthamiana* leaves. P0^YFP^ expression was visualized by confocal microscopy at 3 DAI. P0^YFP^-containing PGs existed in PVA infiltrated leaf samples regardless of the capacity of PVA to replicate or move, showing that neither of these viral processes was required for PG formation. The presented Z-stack projections were acquired using identical imaging settings and image preparation for each panel. Scale bars; 100 μm.
(TIF)

S3 Fig The assembly of P0, UBP1, AGO1 and eIF(iso)4E into PGs is HCpro dependent. (A) P0^YFP^-containing granule-like structures are not present during expression of free RFP whereas they were present during HCpro^RFP^ expression (see Fig 2A), showing that HCpro induces P0 granules. (B) HCpro^RFP^-containing granules did not contain free YFP, verifying that the overlap of P0 and HCpro signals in PGs in Fig 2A is specific. (C) When UPB1^YFP^ was co-expressed with free RFP, UBP1 was mainly localized to the nucleus and the amount of UBP1 granules was small. This demonstrated that HCpro-containing granules recruit UBP1. (D) DCP1^CFP^-containing granules were observed also in the absence of HCpro, but they were not labeled by control RFP. (E) The signal intensity from AGO1^CFP^ was low when co-expressed with free RFP, and no granule structures containing AGO1 could be detected. The increase in the signal intensity observed when AGO1^CFP^ was co-expressed with HCpro^RFP^ (in Fig 2D) compared to expression with free RFP, was consistent with the demonstration that HCpro causes accumulation of specifically AGO1 of *Arabidopsis thaliana* (Chiu M-H, Chen I-H, Baulcombe DC, Tsai C-H. The silencing suppressor P25 of Potato virus X interacts with Argonaute1 and mediates its degradation through the proteasome pathway. Mol Plant Pathol. 2010;11: 641–649. doi:10.1111/j.1364-3703.2010.00634.x). (F) P0^YFP^ and eIF(iso)4E^RFP^ signals overlapped fully in PVA induced PGs. eIF(iso)4E was never observed in granules unless PGs were induced. (G) Bar graph shows the percentage of PGs labeled by eIF(iso)4E^RFP^. PG formation was induced by PVA^CPmut^ in (F) and (G). The confocal images are projections of Z-stacks and acquired with identical imaging settings used for the related images presented in (Fig 2). Overlapping signals are white in the overlay. Scale bars 10 μm. (H—I) The frequency of cells/mm^2^ showing UBP1^YFP^-labelled granules increased upon 30 min heat treatment at 42°C (H), whereas no increase in the frequency of P0^YFP^ labeled PGs could be observed even after 120 min heat stress (I). PVA expression functioned as a positive control for PG induction in (I).
(TIF)

S4 Fig HCpro mutants lacking RNA silencing suppression and eIF4E binding are deficient in PG induction. (A) P0^YFP^ was co-expressed with RFP-tagged HCpro, 4Ebd-HCpro and sd- HCpro and western blot analysis was carried out to detect P0^YFP^ using anti-GFP (upper panel), supporting that P0 only accumulated in the presence of native HCpro capable of PG induction. Detection of the RFP-tagged proteins using anti-RFP revealed different accumulation levels (lower panel). (B) The capacity of RFP-tagged HCpro, 4Ebd-HCpro and sd-HCpro to suppress RNA hairpin-triggered RNA silencing was analyzed. Expression of a *RLUC* RNA hairpin (hpRLUC) reduced RLUC activity derived from co-expressed RLUC (compare GUS to GUS + hpRLUC). Co-expression of HCpro^RFP^ restored RLUC activity fully, 4Ebd-HCpro^RFP^ partially and sd-HCpro^RFP^ hardly at all, verifying that sd-HCpro^RFP^ was truly silencing suppression deficient but also revealing reduced suppression capacity of 4Ebd-HCpro. (C) The same assay used in (B) was applied to verify silencing suppression capacity of applied VSRs. HCpro, p19, 2b and p25 could all restore RLUC activity although to a varying degree in the presence of hpRLUC, while control FLUC activity not targeted by an RNA hairpin silencing remained unaffected (D). Neither P0 nor VPg suppressed hpRLUC-induced silencing (C). Data in (B, C and D) are presented as mean + standard deviation (n = 4).
(TIF)

S5 Fig PGs regulate PVA translation but are insensitive to translational inhibitors. (A-D) FLUC translation remains unaltered by PG components. Neither (A) FLUC activities derived from a transiently expressed non-viral control mRNA nor (B) FLUC mRNA levels were affected by GUS, HCpro, VPg or VPg + HCpro co-expression. These were determined in parallel with PVA RLUC activities and RNA levels (Fig 5E and 5F). (C) FLUC activities derived from a transiently expressed non-viral control mRNA during UBP1 (hpUBP1) and VCS (hpVCS) silencing when co-expressed with GUS or VPg, in parallel with PVA RLUC activities (Fig 5H). The controls were empty silencing vector (hp–) and PDS (hpPDS) silencing. (D) FLUC activities were determined during co-expression of GUS, UBP1 or UBP1rrm, with VPg or GUS, in parallel with the PVA RLUC activities (Fig 5I). The data in (A-D) is presented as mean + standard deviation (n = 4). (E) RNA hairpin-triggered UBP1 and VCS silencing was verified with qPCR. PP2A gene was used to normalize UBP1 and VCS mRNA amounts. (F) An image of isolated protoplast expressing P0^YFP^ and PVA^CPmut^. Due to high variability in number and size of PGs between single cells, no attempt was done to quantify effects of inhibitors on the amount/size of PGs per cell. Scale bar; 50 μm. (G) PG-containing protoplasts were treated with 10 μg ml-1 cycloheximide, actinomycin D or puromycin for 3h and imaged in comparison to non-treated protoplasts. A calculation of the frequency of protoplasts that contained PGs in control or after inhibitor treatments revealed no significant differences between the samples. These results suggest that PGs differ from conventional SGs or PBs. 15 images were analyzed for each treatment and presented as mean + standard deviation. (H) Representative images after inhibitor treatment in (G). Scale bar; 5 μm.
(TIF)

S6 Fig Cell-to-cell spread of infection is not dependent on PG components. (A) GFP-tagged PVA was inoculated using a low *Agrobacterium* density (OD_600_ 0.0005) to initiate infection from single cells. The low cell density in the inoculum resulted in separated PVA infection foci (arrows), which could be visualized by GFP fluorescence using epifluorescence microscopy at 3 days after infiltration. Non-infected tissue (mock) was used as a control for GFP fluorescence. Scale bar; 1 mm. (B) The number of infection foci per mm^2^ was calculated in UBP1-, VCS-, P0-, eIF4E/eIF(iso)4E- and PDS-silenced leaves (n = 16) to determine whether initiation of the infection was compromised under the analyzed conditions. No difference could be detected, showing that infection initiation occurred at comparable rates under these circumstances. (C) At 4 days after infiltration, the infection had spread almost throughout the leaf, and the percentage of infected leaf area was determined during silencing of indicated PG-associated host factors (n = 16). No difference was observed, showing that cell-to-cell movement was not reduced during silencing of PG components. (D) Representative images are shown from the calculation of infected area at 4 DAI presented in (C). Here, the fluorescence intensities are not comparable as images from UBP1, VCS, P0 and eIF4E/eIF(iso)4E silencing were acquired with increased sensitivity. This was done in order to visualize cell-to-cell spread of infection despite decreased fluorescence intensities due to reduced viral gene expression at the cellular level. Non-infected tissue was used to verify that the signals during also increased sensitivity were derived from GFP. Scale bar; 1 mm. (E) TRV-induced UBP1 and VCS silencing was quantified by RT-qPCR. PP2A mRNA was used to normalize UBP1 and VCS mRNA amounts.
(TIF)
