# Supplementary figures and images for: Formation of Potato Virus A-Induced RNA Granules and Viral Translation Are Interrelated Processes Required for Optimal Virus Accumulation
Source: PLoS Pathog. 2015 Dec 7;11(12):e1005314. doi: 10.1371/journal.ppat.1005314 (PMC4671561; doi:10.1371/journal.ppat.1005314)

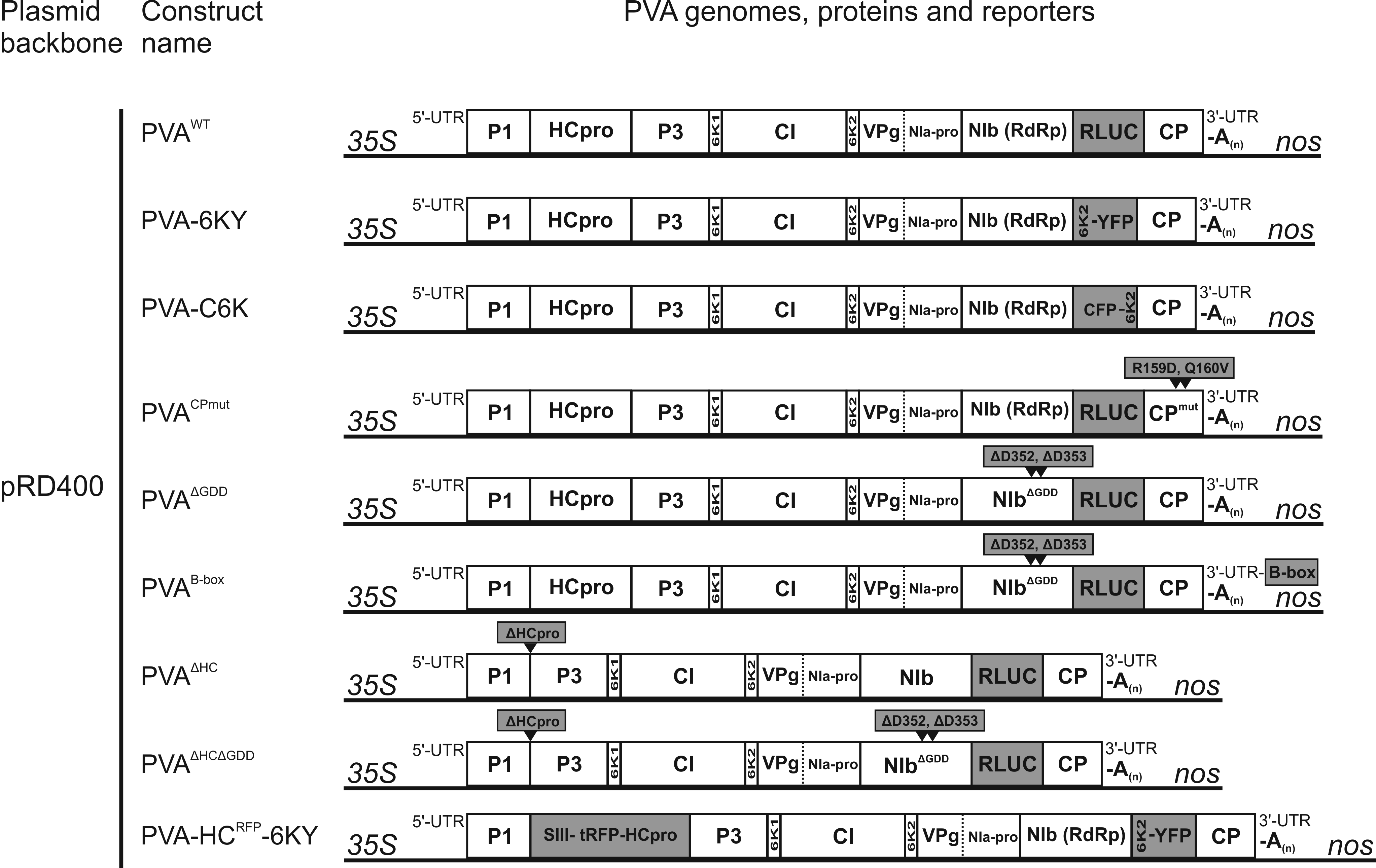

Supplement: S1 Fig — Renilla luciferase (RLUC) inserted into the PVA icDNA between RNA-dependent RNA polymerase (NIb) and coat protein (CP) genes [16]. PVACPmut and PVAΔGDD represent movement- and replication-deficient Rluc-tagged PVAs, respectively (mutated amino acids indicated) [16]. PVA-6KY and PVA-C6K are PVAs modified with an extra copy of 6K2-YFP or Cerulean FP-6K2 gene, respectively. PVAΔHC is PVA virus construct with a deleted HCpro gene. PVAΔHCΔGDD is PVAΔGDD having HCpro gene deleted and PVAB-box has B-box RNA element fused to the 3’UTR of PVAΔGDD. PVA-HCRFP-6KY has HCpro fused to RFP and StrepIII-tag in addition to the extra 6K2-YFP. (TIF) [file ppat.1005314.s001.tif]

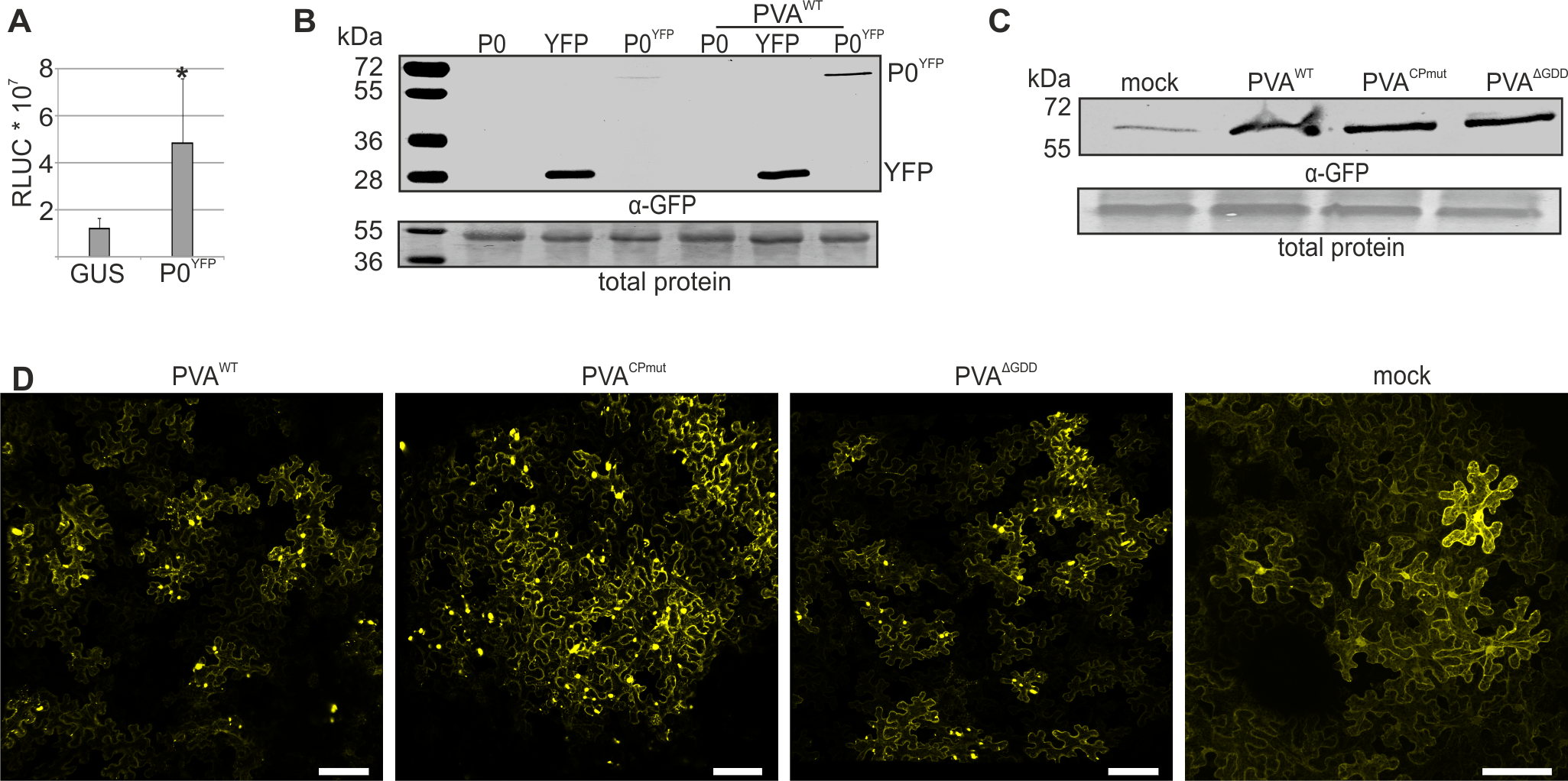

Supplement: S2 Fig — (A) GUS (control) and P0YFP were co-expressed with RLUC-tagged PVAWT and viral RLUC activity was analyzed at 7 days after infection (DAI). To mimic natural infection process PVA was inoculated using a low Agrobacterium density (OD600 0.0005) to initiate infection from single cells from which the infection then spreads via cell-to-cell movement (as in S6 Fig). RLUC activity reporting for the activity of viral gene expression is presented as mean + standard deviation and a student’s t-test shows that viral RLUC activity is significantly enhanced in P0YFP samples when compared to the control samples (p < 0.05 *), showing that P0YFP promotes PVA gene expression similarly to non-fused P0 [15]. This validated the use of fusion protein P0YFP in studying the role of P0 in PVA infection. (B) P0, YFP and P0YFP were expressed in the presence and absence of PVAWT. YFP and P0YFP accumulation was detected by anti-GFP western blot analysis. The non-tagged P0 served as a negative control for YFP detection. P0YFP, but not YFP, accumulation was enhanced in the presence of PVA, suggesting that PVA does not cause accumulation of any co-expressed protein but rather it is P0 specific response. The membrane was also stained for total protein using Ponceau S to verify loading accuracy and is shown at the position of the large RuBisCO subunit. (C) P0YFP expression was analyzed, in parallel with confocal microscopy imaging in (D), by western blotting using anti-GFP antibody. The membrane was also stained for total protein using Ponceau S to verify loading and is shown at the position of the large RuBisCO subunit. Correlating with PG-formation in (D), P0YFP accumulation was enhanced in the presence of all PVA variants compared to the non-infected mock sample. (D) P0YFP was expressed together with PVAWT, PVACPmut, PVA∆GDD or GUS (mock) using Agrobacterium-mediated transformation of N. benthamiana leaves. P0YFP expression was visualized by confocal microscopy at 3 DAI. P0YFP-containing PGs exis [file ppat.1005314.s002.tif]

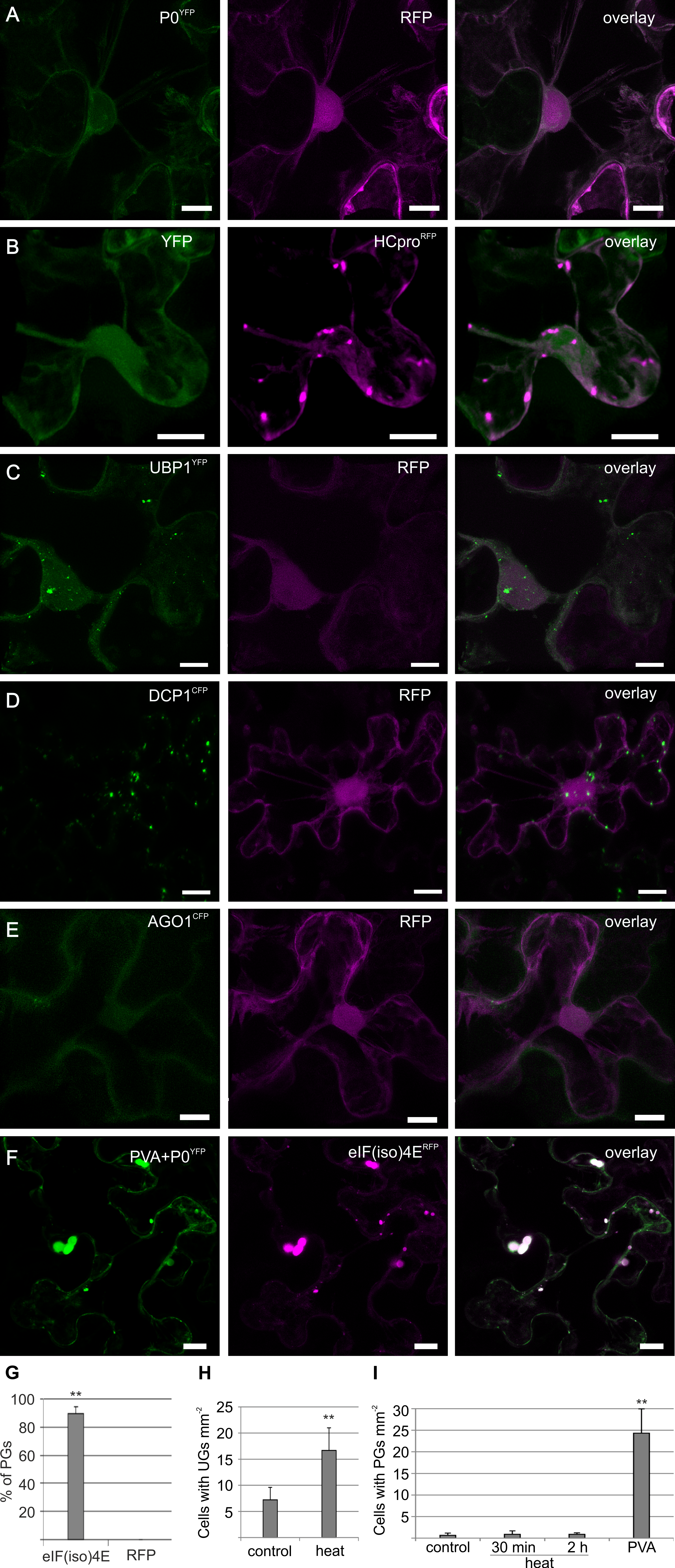

Supplement: S3 Fig — (A) P0YFP-containing granule-like structures are not present during expression of free RFP whereas they were present during HCproRFP expression (see Fig 2A), showing that HCpro induces P0 granules. (B) HCproRFP-containing granules did not contain free YFP, verifying that the overlap of P0 and HCpro signals in PGs in Fig 2A is specific. (C) When UPB1YFP was co-expressed with free RFP, UBP1 was mainly localized to the nucleus and the amount of UBP1 granules was small. This demonstrated that HCpro-containing granules recruit UBP1. (D) DCP1CFP-containing granules were observed also in the absence of HCpro, but they were not labeled by control RFP. (E) The signal intensity from AGO1CFP was low when co-expressed with free RFP, and no granule structures containing AGO1 could be detected. The increase in the signal intensity observed when AGO1CFP was co-expressed with HCproRFP (in Fig 2D) compared to expression with free RFP, was consistent with the demonstration that HCpro causes accumulation of specifically AGO1 of Arabidopsis thaliana (Chiu M-H, Chen I-H, Baulcombe DC, Tsai C-H. The silencing suppressor P25 of Potato virus X interacts with Argonaute1 and mediates its degradation through the proteasome pathway. Mol Plant Pathol. 2010;11: 641–649. doi:10.1111/j.1364-3703.2010.00634.x). (F) P0YFP and eIF(iso)4ERFP signals overlapped fully in PVA induced PGs. eIF(iso)4E was never observed in granules unless PGs were induced. (G) Bar graph shows the percentage of PGs labeled by eIF(iso)4ERFP. PG formation was induced by PVACPmut in (F) and (G). The confocal images are projections of Z-stacks and acquired with identical imaging settings used for the related images presented in (Fig 2). Overlapping signals are white in the overlay. Scale bars 10 μm. (H—I) The frequency of cells/mm2 showing UBP1YFP-labelled granules increased upon 30 min heat treatment at 42°C (H), whereas no increase in the frequency of P0YFP labeled PGs could be observed even after 120 min heat stress (I). PVA [file ppat.1005314.s003.tif]

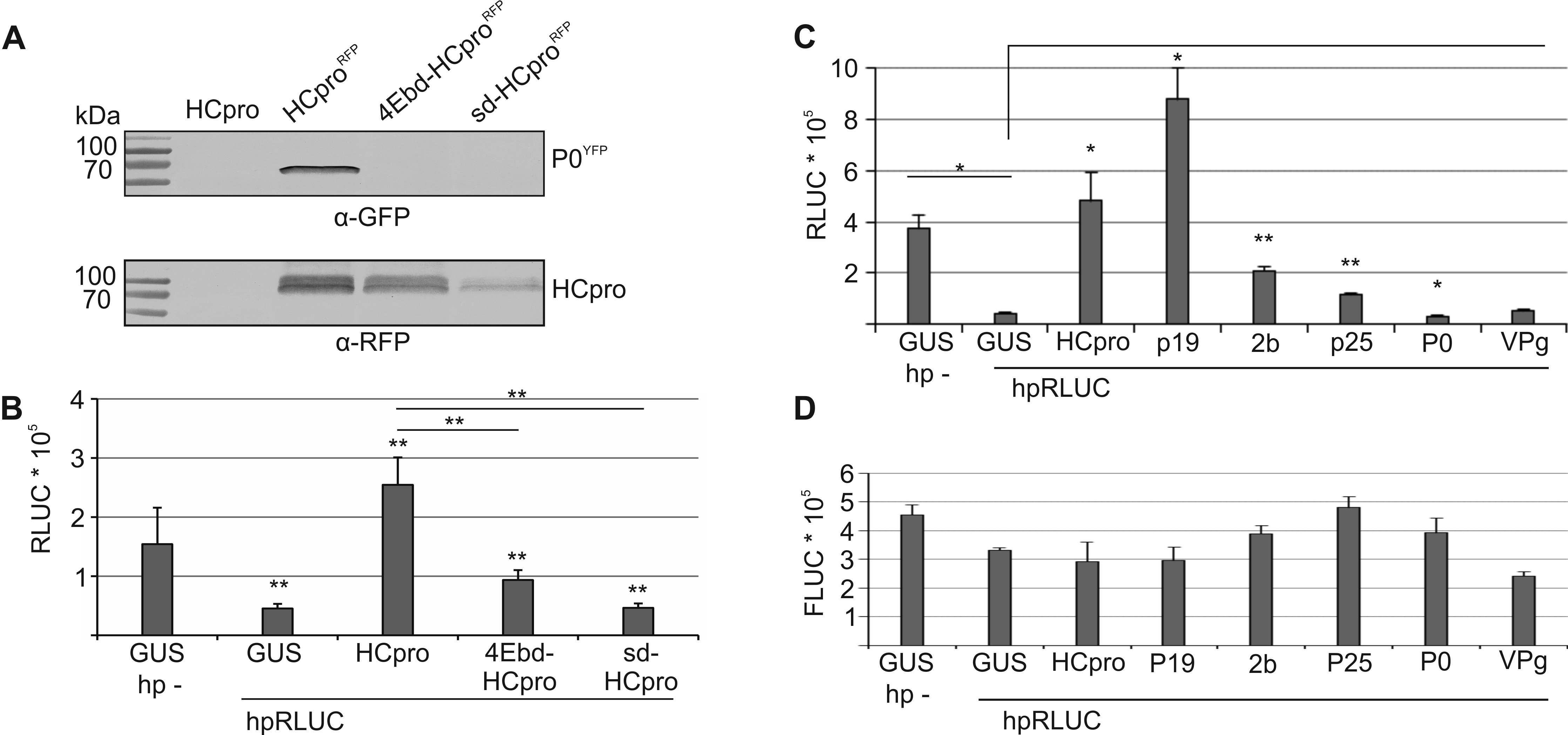

Supplement: S4 Fig — (A) P0YFP was co-expressed with RFP-tagged HCpro, 4Ebd-HCpro and sd- HCpro and western blot analysis was carried out to detect P0YFP using anti-GFP (upper panel), supporting that P0 only accumulated in the presence of native HCpro capable of PG induction. Detection of the RFP-tagged proteins using anti-RFP revealed different accumulation levels (lower panel). (B) The capacity of RFP-tagged HCpro, 4Ebd-HCpro and sd-HCpro to suppress RNA hairpin-triggered RNA silencing was analyzed. Expression of a RLUC RNA hairpin (hpRLUC) reduced RLUC activity derived from co-expressed RLUC (compare GUS to GUS + hpRLUC). Co-expression of HCproRFP restored RLUC activity fully, 4Ebd-HCproRFP partially and sd-HCproRFP hardly at all, verifying that sd-HCproRFP was truly silencing suppression deficient but also revealing reduced suppression capacity of 4Ebd-HCpro. (C) The same assay used in (B) was applied to verify silencing suppression capacity of applied VSRs. HCpro, p19, 2b and p25 could all restore RLUC activity although to a varying degree in the presence of hpRLUC, while control FLUC activity not targeted by an RNA hairpin silencing remained unaffected (D). Neither P0 nor VPg suppressed hpRLUC-induced silencing (C). Data in (B, C and D) are presented as mean + standard deviation (n = 4). (TIF) [file ppat.1005314.s004.tif]

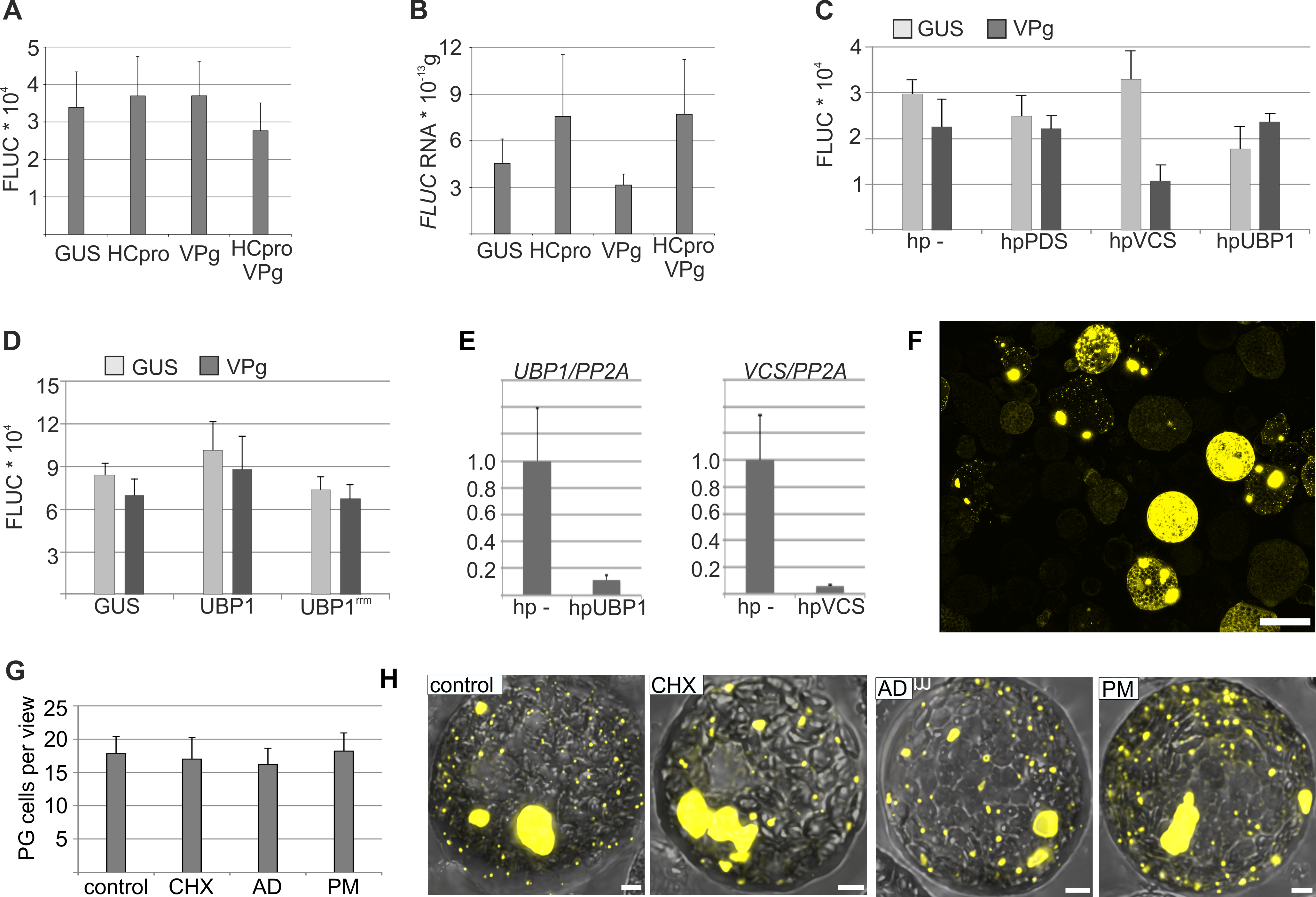

Supplement: S5 Fig — (A-D) FLUC translation remains unaltered by PG components. Neither (A) FLUC activities derived from a transiently expressed non-viral control mRNA nor (B) FLUC mRNA levels were affected by GUS, HCpro, VPg or VPg + HCpro co-expression. These were determined in parallel with PVA RLUC activities and RNA levels (Fig 5E and 5F). (C) FLUC activities derived from a transiently expressed non-viral control mRNA during UBP1 (hpUBP1) and VCS (hpVCS) silencing when co-expressed with GUS or VPg, in parallel with PVA RLUC activities (Fig 5H). The controls were empty silencing vector (hp–) and PDS (hpPDS) silencing. (D) FLUC activities were determined during co-expression of GUS, UBP1 or UBP1rrm, with VPg or GUS, in parallel with the PVA RLUC activities (Fig 5I). The data in (A-D) is presented as mean + standard deviation (n = 4). (E) RNA hairpin-triggered UBP1 and VCS silencing was verified with qPCR. PP2A gene was used to normalize UBP1 and VCS mRNA amounts. (F) An image of isolated protoplast expressing P0YFP and PVACPmut. Due to high variability in number and size of PGs between single cells, no attempt was done to quantify effects of inhibitors on the amount/size of PGs per cell. Scale bar; 50 μm. (G) PG-containing protoplasts were treated with 10 μg ml-1 cycloheximide, actinomycin D or puromycin for 3h and imaged in comparison to non-treated protoplasts. A calculation of the frequency of protoplasts that contained PGs in control or after inhibitor treatments revealed no significant differences between the samples. These results suggest that PGs differ from conventional SGs or PBs. 15 images were analyzed for each treatment and presented as mean + standard deviation. (H) Representative images after inhibitor treatment in (G). Scale bar; 5 μm. (TIF) [file ppat.1005314.s005.tif]

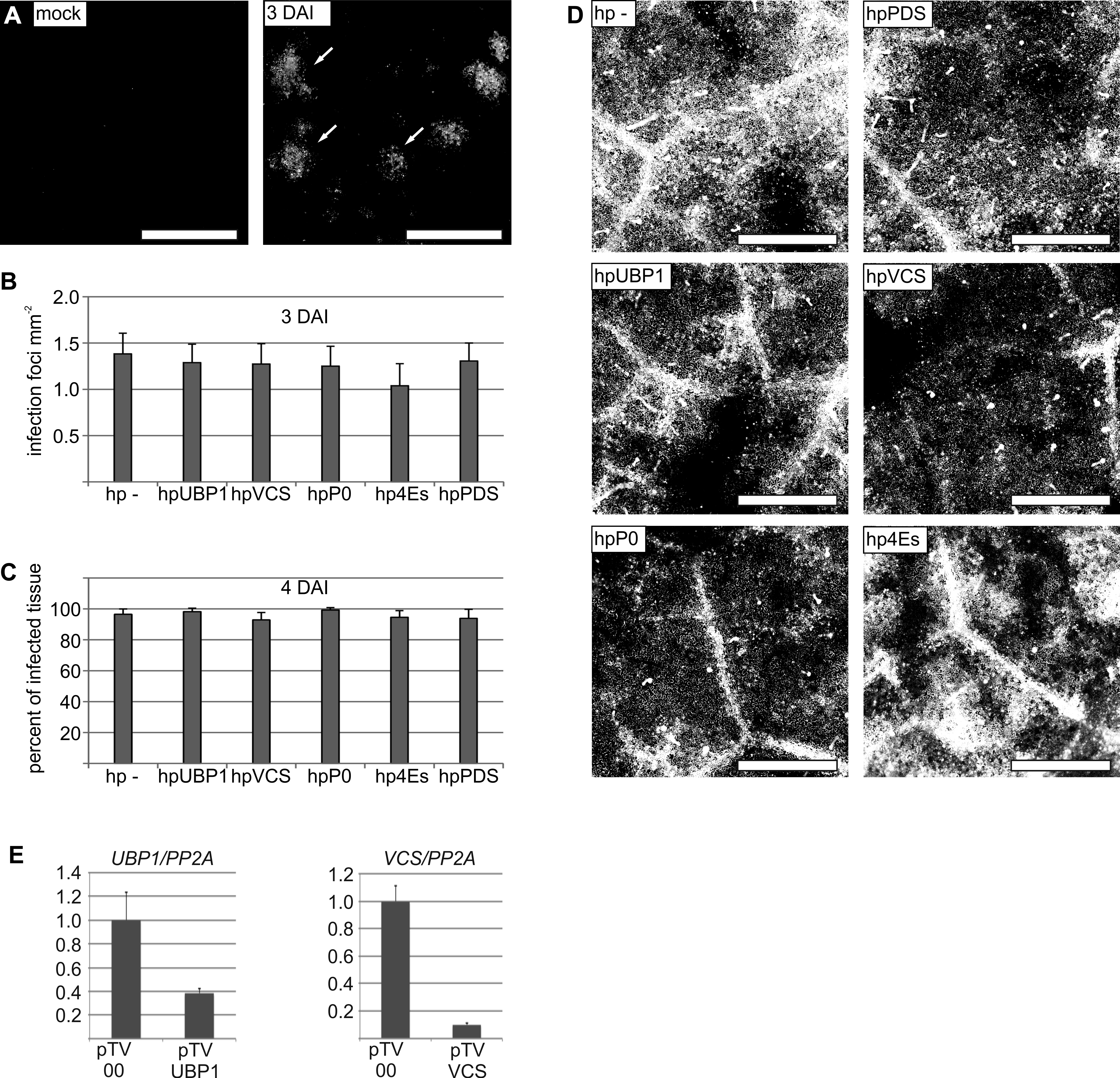

Supplement: S6 Fig — (A) GFP-tagged PVA was inoculated using a low Agrobacterium density (OD600 0.0005) to initiate infection from single cells. The low cell density in the inoculum resulted in separated PVA infection foci (arrows), which could be visualized by GFP fluorescence using epifluorescence microscopy at 3 days after infiltration. Non-infected tissue (mock) was used as a control for GFP fluorescence. Scale bar; 1 mm. (B) The number of infection foci per mm2 was calculated in UBP1-, VCS-, P0-, eIF4E/eIF(iso)4E- and PDS-silenced leaves (n = 16) to determine whether initiation of the infection was compromised under the analyzed conditions. No difference could be detected, showing that infection initiation occurred at comparable rates under these circumstances. (C) At 4 days after infiltration, the infection had spread almost throughout the leaf, and the percentage of infected leaf area was determined during silencing of indicated PG-associated host factors (n = 16). No difference was observed, showing that cell-to-cell movement was not reduced during silencing of PG components. (D) Representative images are shown from the calculation of infected area at 4 DAI presented in (C). Here, the fluorescence intensities are not comparable as images from UBP1, VCS, P0 and eIF4E/eIF(iso)4E silencing were acquired with increased sensitivity. This was done in order to visualize cell-to-cell spread of infection despite decreased fluorescence intensities due to reduced viral gene expression at the cellular level. Non-infected tissue was used to verify that the signals during also increased sensitivity were derived from GFP. Scale bar; 1 mm. (E) TRV-induced UBP1 and VCS silencing was quantified by RT-qPCR. PP2A mRNA was used to normalize UBP1 and VCS mRNA amounts. (TIF) [file ppat.1005314.s006.tif]
